# Going with the flow: Intraspecific variation may act as a natural ally to counterbalance the impacts of global change for the riparian species *Populus deltoides*

**DOI:** 10.1111/eva.12854

**Published:** 2019-09-20

**Authors:** Julie Godbout, Marie‐Claude Gros-Louis, Manuel Lamothe, Nathalie Isabel

**Affiliations:** ^1^ Ministère des Forêts, de la Faune et des Parcs, Direction de la recherche forestière Québec QC Canada; ^2^ Canadian Forest Service, Laurentian Forestry Centre Natural Resources Canada Québec QC Canada

**Keywords:** adaptive capacity, connectivity, conservation, gene flow, global change, host–pathogen interactions, intraspecific differentiation, *Populus*

## Abstract

The speed and magnitude of global change will have major impacts on riparian ecosystems, thereby leading to greater forest vulnerability. Assessing species’ adaptive capacities to provide relevant information for vulnerability assessments remains challenging, especially for nonmodel species like the North American *Populus deltoides* W. Bartram ex Marshall. The objective of this study was to understand how genomic diversity of this foundation species was shaped by its environment (climate, soil, and biotic interactions) to gauge its adaptive capacity. We used two complementary approaches to get a full portrait of *P. deltoides* genetic diversity at both the species and whole‐genome ranges. First, we used a set of 93 nuclear and three chloroplastic SNP markers in 946 individuals covering most of the species' natural distribution. Then, to measure the degree of intraspecific divergence at the whole‐genome level and to support the outlier and genomic‐environment association analyses, we used a sequence capture approach on DNA pools. Three distinct lineages for *P. deltoides* were detected, and their current distribution was associated with abiotic and biotic variations. The comparison between both cpDNA and ncDNA patterns showed that gene flow between the lineages is unbalanced. The southern and northeastern populations may benefit from the input, through river flow, of novel alleles located upstream to their local gene pools. These alleles could migrate from populations that are already adapted to conditions that fit the predicted climates in the receiving local populations, hotter at the northeastern limit and drier in the Central United States. These “preadapted” incoming alleles may help to cope with maladaptation in populations facing changing conditions.

## 1 INTRODUCTION

The anticipated rate and magnitude of global change will have major impacts on boreal and temperate forest ecosystems. Recent findings suggest that rapid climate shifts may outpace existing compensatory mechanisms of tree species, such as physiological acclimation, leading to greater forest vulnerability and tree mortality (Allen et al., 2010). Given the speed of these changes (IPCC, 2018; Kemp, Eichenseer, & Kiessling, 2015), it is unlikely that the ability of species to adjust to the changing environment will come from new favorable mutations because these processes are very slow (Orr & Unckless, 2008). Yet, considerable uncertainties remain as to the amplitude of adaptive capacity (genetic makeup and plasticity) to abiotic and biotic stresses, alone or in combination, in long‐lived species. To date, climate change vulnerability assessments have been conducted using climate envelopes without considering the genetic variability although this trend has started to change (Bothwell et al., 2017; Jones, Watson, Possingham, & Klein, 2016; Wan et al., 2014). Indeed, the vulnerability of forest ecosystems to global change at regional scales will not only depend on the magnitude of the environmental change, but also on the adaptive capacity of existing tree species, which could be enhanced through forest management practices and other adaptation measures.

Assessing the adaptive capacity of a species to provide relevant information that fits within a vulnerability framework remains challenging, especially for long‐lived species (for a review, see Gauthier et al., 2014). The evaluation of phenotypes in common garden or reciprocal transplant experiments in combination with genomics may produce valuable information, such as species‐specific sensitivity to environmental factors (Housset et al., 2018). For long‐lived species, these long‐term field tests were mostly conducted using commercial species and they generally did not cover the entire species range because they were established in the early 70s, that is, before climate change concerns were raised. There is therefore an urgent need to gauge the adaptive capacity of “less studied” species to provide information for vulnerability assessment frameworks that prioritize and foster adaptation strategies. One option to consider consists of getting a portrait of the genomic diversity across taxa ranges (Aubin et al., 2016).

The genomic diversity of a species results from the combination of neutral (genetic drift, migration, and mutation) and selective evolutionary processes. Thus, identifying the main abiotic and biotic factors that have influenced genomic diversity and the way genes are exchanged between populations may be one of the first steps to take when characterizing the ability of taxa to persist in a changing environment. Species with wide distributions across varying climates and environments may help elucidate how genetic makeup is shaped and determine the main underlying factors influencing this makeup.

There is a pressing issue surrounding the decline of foundation species like riparian eastern cottonwood (*Populus deltoides* W. Bartram ex Marshall) that constitute the structural basis of vital ecosystems (Johnson & Haight, 1984; Rood et al., 2005). Such declines could lead to the disruption of fundamental ecosystem processes. *P. deltoides*, one of the largest hardwoods in eastern North America (Little, 1979), is found in transitional zones between land and rivers, and, as such, it provides many ecosystem services. These forests act as natural water biofilters and protect riparian environments from excessive sedimentation, polluted surface runoff, and erosion. They supply shelter and food for many terrestrial and aquatic animals (Finch & Ruggiero, 1993; Knopf, Johnson, Rich, Samson, & Szaro, 1988), and, when considering them on a broader scale, they form a forested network that assures connectivity for associated communities.

The eastern cottonwood has been subjected to major range modifications in its ancient and more recent history. During the last glaciation, the northern part of its range was covered by ice, so populations from these regions reached this area only 10,000 years ago, once the ice had retreated (Dyke et al., 2002). Over the last few centuries, since the first European settlements were established, the colonization of river banks and the construction of dams to serve agricultural water needs have resulted in the decline of cottonwood stands in many drainage basins (Dixon, Johnson, Scott, Bowen, & Rabbe, 2012; Rood, Braatne, & Hughes, 2003). Hence, northern populations of eastern cottonwood may have been impacted by several bottleneck events so that they may now present a lower genetic diversity, which might challenge their capacity to adapt to present and future changes in climate. Climate models predict a northward expansion for *P. deltoides* (Morin & Thuiller, 2009). Hence, we could suppose that these relatively new northern populations will be, once again, the sources for northward migrations. Given these facts, particular attention has been given to the northern part of the *P. deltoides* range in this study.

Evolutionary processes such as hybridization and speciation have shaped the genomic diversity of species, and the study of ongoing ecological speciation processes may help to understand what drives differentiation (Rundle & Nosil, 2005; Schluter, 2001). Members of the Salicaceae family are prone to hybridization (Ellstrand, Whitkus, & Rieseberg, 1996; Karrenberg, Edwards, & Kollmann, 2002), which makes *Populus* species useful models when trying to understand the role of hybridization in adaptation. The classification of the *Populus* species is, at best, challenging and not always compatible with the dichotomous “tree of life” metaphor (Hamzeh & Dayanandan, 2004; Kersten et al., 2016), which may be indicative of extensive gene flow during and after speciation. Like many *Populus* species, intraspecific phenotypic variation is observed in *P. deltoides*, which translates into the delineation of distinct subspecies (Eckenwalder, 1977, 1996; Haverbeke, 1990). One could suggest that *Populus* species are highly “reactive” to ecological changes. Such differentiation provides the opportunity to study how the environment might have influenced the resulting genomic diversity (Christe et al., 2016; Dewoody, Trewin, & Taylor, 2015; Joseph & Lexer, 2008; Lexer, Fay, Joseph, Nica, & Heinze, 2005; Meirmans, Godbout, Lamothe, Thompson, & Isabel, 2017; Suarez‐Gonzalez et al., 2016). The two most widespread subspecies of *P. deltoides*, subsp. *deltoides*, and subsp. *monilifera*, respectively, cover most of the northwestern and southeastern part of its range, while the subsp. *wislizeni* is found in a more restricted area in the southwest (Figure S1). The *P. deltoides* range covers a vast territory, so populations are found in a large variety of abiotic (climatic, but also edaphic) and biotic conditions. We could thus hypothesize that such a vast range of environmental variations would translate into different adaptations resulting from the selection of different alleles across the species’ range.

*Populus deltoides* occurs along rivers and does not form continuous forests, so gene flow between populations (connectivity) may be restricted by this unidimensional aspect of riparian forests. Moreover, their riparian setting means that they are subjected to a regime of frequent disturbances caused by floods (Braatne, Rood, & Heilman, 1996). This would suggest that their recipe for success consists of a combination of plasticity and agility (i.e., capacity for rapid evolutionary change). At the population scale, this capacity to react promptly and seize opportunities may be associated with access to sources of novelty. Indeed, existing genetic variation can be increased by an input from other taxa, either other species or ecotypes in order to “evolutionarily rescue” local populations that may face maladaptation under climate change (Gonzalez, Ronce, Ferriere, & Hochberg, 2013). Under this scenario, new gene arrivals may provide useful material to cope with new climate and soil conditions, but could also help thwart local pathogens. Hence, populations as whole networks may contribute to an increased resilience at the species level; some populations can benefit from a steady input of alleles from other populations that, if useful (i.e, provide an advantage in the new conditions), will be incorporated into the local gene pool via adaptive introgression (see a selection of reviews about this concept here: Hamilton & Miller, 2016; Hedrick, 2013; Pfennig, Kelly, & Pierce, 2016).

We pursued two objectives in this study. First, we wanted a portrait of the population structure and genomic diversity of *P. deltoides* across its range, with particular interest in its northern boundary. Our hypothesis was that genomic differentiation fits the taxonomical subdivision in this species. In addition, we were interested in gaining a better understanding of population connectivity at different scales, that is, how gene exchange generally occurs between populations, as well as between and within subspecies, given the landscape heterogeneity in which populations are found. Our second objective was to determine the main environmental (abiotic and biotic) factors that influence the genomic diversity of *P. deltoides*. We used four sets of environmental data: geographic, climatic, edaphic, and biotic variables. For the latter, we used genomic data from *Sphaerulina musiva, P. deltoides*'s associated fungal pathogen, that causes leaf spots in natural stands. We compare the effect of each set of variables on the diversity of *P. deltoides* and identify those that best explain the differentiation observed between populations. This characterization will provide information for a global change vulnerability assessment, which in turn helps prioritize adaptation measures, in terms of forest and land management, for this foundation species.

## 2 MATERIAL AND METHODS

### 2.1 Sampling and DNA extraction

**Figure 1:**
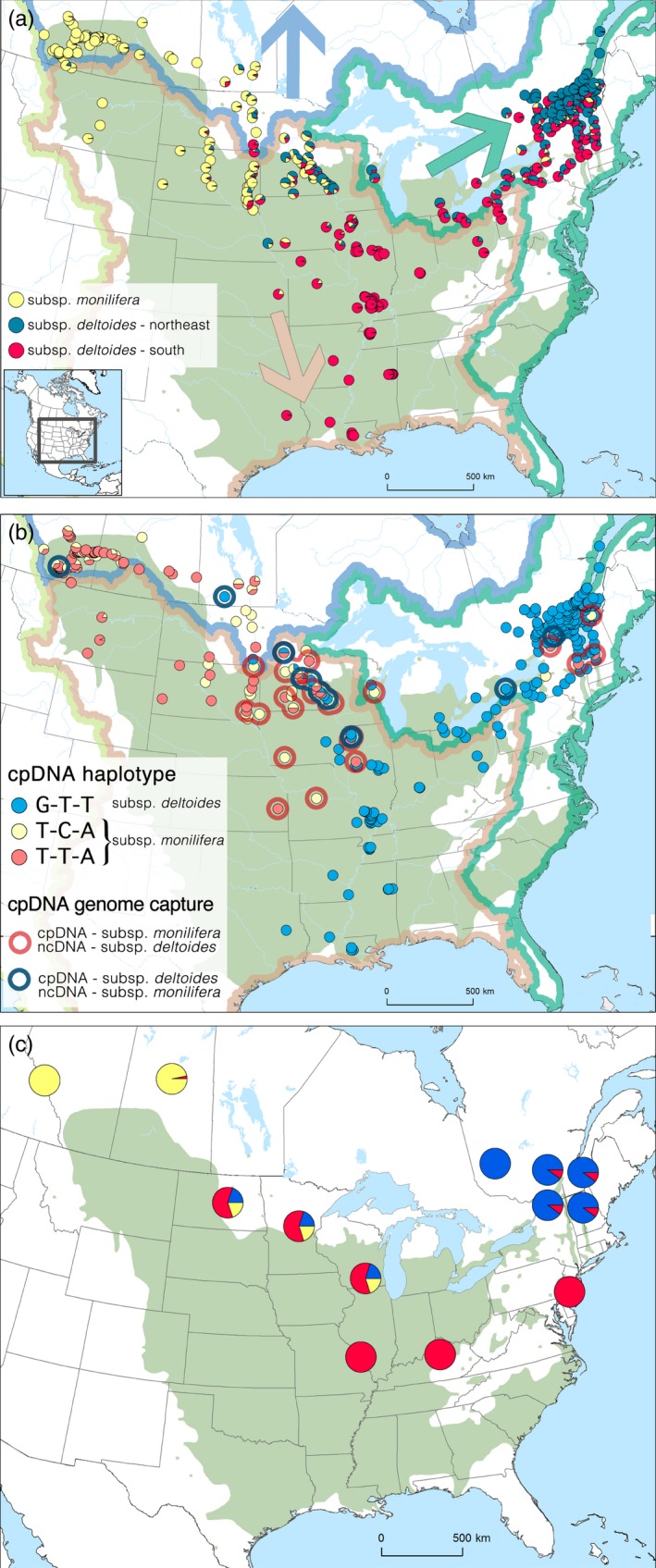
(a) Structure results for a *K* = 3 partition. Green color on map corresponds to the natural range of *Populus deltoides* according to Little (1971). Thick lines represent the delineation of the ocean watersheds areas: blue: Arctic, green: Atlantic, light red: Caribbean, and light green: Pacific. Colored arrows show the direction of river flow within each drainage basin. (b) Geographical distribution of the three chloroplast haplotypes. Circled populations indicate a capture event between the two subspecies where blue shows trees presenting a *monilifera* subsp. background with a *deltoides* subsp. chloroplast and red circles correspond to trees presenting a *deltoides* subsp. nuclear signature with *monilifera* subsp. chloroplast DNA. (c) Bayesian clustering results for *P. deltoides* native fungal pathogen (draw according Sakalidis et al., 2016)

Material from 1,004 trees covering most of the *P. deltoides* natural range was obtained from various sources or sampled in natural stands (Figure 1). Many collaborators provided georeferenced samples that ranged from 42° to 52°N latitude and 69° to 114°W longitude. DNA was extracted from dried leaf material using the Nucleospin 96 Plant II kit (Macherey‐Nagel) following the manufacturer's protocol for vacuum processing with the following modifications: (a) cell lysis using buffers PL2 and PL3, PL2 was heated for 2 hr at 65 °C instead of 30 min and (b) elution with an in‐house Tris–Cl 0.01 mM pH 8.0 buffer.

### 2.2 Sequencing and marker development

#### 2.2.1 Nuclear SNP markers

Nuclear SNPs were developed from DNA sequences obtained for 20 selected individuals of *P. deltoides* distributed throughout the species natural range using a pool sequencing approach similar to the one presented by Isabel, Lamothe, and Thompson (2013). More than 35 million Solexa 100 bp paired‐end reads were obtained and aligned to the *P. trichocarpa* genome reference v.2.0 (Tuskan et al., 2006) (*Populus trichocarpa* v2.0, http:://www.phytozome.net/poplar) using CLC Genomic Workbench 7.5.1 (QIAGEN). We selected the biallelic SNP variants in regions presenting a minimum coverage of 15 individuals out of 20. We tried to choose SNPs that were in the same genes as those used in a previous study focusing on the intraspecific diversity of *Populus balsamifera* in order to be able to compare the two species (Meirmans et al., 2017). These SNPs were located in genes potentially involved in host–pathogen interactions (Azaiez, Boyle, Levée, & Séguin, 2009; Levée et al., 2009). These genes are implicated in primary and secondary metabolic processes, defense and stress‐related mechanisms, or cell‐wall composition and lignification. In total, 102 intraspecific SNPs were used to build three Sequenom iPlex Gold genotyping arrays (Agena Bioscience).

#### 2.2.2 Chloroplast SNP markers

Chloroplastic SNPs were essentially chosen from genes where coverage was no <1,000× using the same approach as presented in Meirmans et al. (2017). The main objective was to select SNPs having the most diverse allele frequencies to get information that is as uncorrelated as possible. In total, 12 SNPs with a minor allele frequency ranging between 0.10 and 0.28 were selected.

#### 2.2.3 Genotyping

Specifically, a total of 102 nuclear and 12 chloroplastic SNPs were chosen for inclusion in a Sequenom iPLEX MassARRAY genotyping assay. An optimized set of primers for multiplex PCR was designed in the invariant flanking regions (150 pb) of our SNP by the McGill University and Génome Québec Innovation Centre (Montréal, Canada) using internal protocols. Using the 114 SNPs, three different iPlex Gold assays (Sequenom) were built with 34, 40, and 40 SNPs, respectively. The 1,004 *P. deltoides* samples were then genotyped using these genotyping assays. To discard hybrids, the samples were also genotyped with an assay of 36 interspecific SNPs (Isabel et al., 2013) designed to identify poplar species and their early‐generation hybrids in both the Aigeiros and Tacamahaca taxonomic sections, including hybrids used as ornamentals. Moreover, prior to analysis, we used the Genodive software (Meirmans & Van Tienderen, 2004) to eliminate all putative clones from our datasets.

### 2.3 Population structure analysis

We used Structure software (Pritchard, Stephens, & Donnelly, 2000) to analyze the population structure for the entire sample size. Individuals with more than 10% missing data were discarded. Structure version 2.3.4 was used with the admixture model, using the default parameters and without using any prior population information. The MCMC process in Structure ran for 100,000 steps after a burn‐in period of 10,000 steps. Ten repeat analyses were conducted for each *K* value ranging from 1 to 10. The optimal number of clusters was estimated using the Delta *K* statistic (Evanno, Regnaut, & Goudet, 2005) using the Structure Harvester online program (Earl & vonHoldt, 2012). To compare with the Structure outputs and, especially, to confirm or not the most plausible number of clusters, a principal component analysis (PCA) using the “rda” function in the vegan R package (Oksanen et al., 2016) was also performed. We coded the genotypes with respect to the number of alleles (0, 1, or 2). Because both the Structure and PCA methods are sensitive to unbalanced sampling and may misevaluate the *K* number, we used the alternative ancestry prior parameter (i.e., “individual Alpha for each population”) as suggested by Wang (2017) in an additional Structure analysis and we conducted PCA on 20 random subsamples (*n* = 150–200).

Following population structure analyses, we calculated diversity and differentiation indices for all samples and for the different clusters (identified by both Structure and PCA analyses) using the Fstat software (Goudet, 1995). A hierarchical analysis of molecular variance (AMOVA; Excoffier, Smouse, and Quattro (1992)) was used to compare the patterns of population differentiation among all samples and between the groups identified by the population structure analyses. We calculated the diversity and differentiation indices and performed the AMOVA analyses using populations with more than three trees and, for the cluster comparison analyses, using individuals having a Structure *Q*‐value >0.667 (analyses using a *Q*‐value threshold of 0.9 gave very similar results, data not shown).

An outlier test was performed to see if some of the 93 nuclear SNP loci are putatively under selection. For this analysis, we used the Fdist2 function implemented in Arlequin v. 3.5.2 (Excoffier & Lischer, 2010). We used the “hierarchical” option that takes population structure into account to avoid a bias (Excoffier, Hofer, & Foll, 2009) that could be induced by the strong geographical structure of our data. To account for the effect of sampling and minimum population sizes on the Fdist2 analysis, we tested several data configurations. It was thus performed using two different cluster assignments: Structure *Q*‐value thresholds of 0.667 and of 0.9, and we also tested population groupings using populations with more than three trees and more than five trees. Analyses were conducted over the three clusters and by comparing all pairs of clusters. To optimize the number of individuals analyzed for paired analysis, datasets with populations of more than three trees were used. The Fdist2 simulations were performed assuming three genetic groups consisting of 100 populations each. Significance was tested using 20,000 simulations and corrected for multiple testing using a false discovery rate of 0.05 (Benjamini & Hochberg, 1995).

### 2.4 Environmental data

Four types of environmental data were used for this study, three abiotic (climate, soil, and geography, although geography cannot strictly qualify as environmental) and one biotic (pathogen genomic variation). In addition, in order to visually compare the geographic distribution of *P. deltoides* genomic diversity with the drainage basin, we used data representing the ocean watersheds limits in North America (Government of Canada, 2010).

#### 2.4.1 Abiotic factors

Climatic variables covering the 1948–2010 period (75 seasonal and annual variables) were obtained using ClimateNA software v5.21 (Wang, Hamann, Spittlehouse, & Murdock, 2012). Seasonal and annual 62‐year means and standard deviations were calculated for the climatic variables. Soil characteristics (28 variables) were extracted from the Harmonized Global Soil database v.1.2 (Wieder, Boehnert, Bonan, & Langseth, 2014) for each tree location. We used geographical coordinates and elevation from sample locations as geographical variables.

One of each pair of geographic, climatic, and soil variable with correlation coefficients >.7 was removed for most analyses to eliminate redundant variables. Twenty‐six variables were retained: five climatic means, 13 climatic standard deviations, and eight soil variables (see Table 1 for a description and Appendix [Link], [Link] for maps presenting all variables).

**Table 1:** Noncorrelated environmental variables and significant LFMM genomic‐environment association results for the different sampling configurations. In parenthesis, putative gene name, *Populus trichocarpa* gene name, and chromosome number on which the locus is found

Variable type	Variable code	Variable name	Variable description	SNP from LFMM (whole sampling)	SNP from LFMM (subsp. *monilifera*)	SNP from LFMM (subsp. *deltoides*)
Climate (average)	Mean01	Moy_RH_wt	Winter—mean annual relative humidity (%)			K008 (WRKY4, Potri.006G224100, Chr06)
	Mean02	Moy_MAT	Mean annual temperature (°C),			L016 p value450‐1, Potri.005G153800, Chr05)
	Mean03	Moy_TD	Mean temperature difference between mean warmest month temperature and mean coldest month temperature, or continentality (°C),	K008 (WRKY4, Potri.006G224100, Chr6), L016 (P450‐1, Potri.005G153800, Chr05), L031 (HDZIP1, Potri.014G075200, Chr14)		
	Mean04	Moy_MAP	Mean annual precipitation (mm)	J026 (WRKY1, Potri.014G096200, Chr14) L031 (HDZIP1, Potri.014G075200, Chr14)	L037 (PLAC8, Potri.016G087200, Chr16)	
	Mean05	Moy_PAS	Precipitation as snow (mm) between August in previous year and July in current year			
Climate (Standard deviation)	SD01	SD_Tmax_sm	Summer mean maximum temperature (°C)	J026 (WRKY1, Potri.014G096200, Chr14)		
	SD02	SD_Tmin_sm	Summer mean minimum temperature (°C)			
	SD03	SD_PPT_sm	Summer precipitation (mm)			
	SD04	SD_DD5_sm	Summer degree‐days above 5 °C			
	SD05	SD_NFFD_sp	The number of frost‐free days	L015 (FBOX2, Potri.005G170000, Chr5)		
	SD06	SD_Eref_at	Autumn Hargreaves reference evaporation (mm)			
	SD07	SD_CMD_wt	Winter Hargreaves climatic moisture deficit (mm)			
	SD08	SD_CMD_sp	Spring Hargreaves climatic moisture deficit (mm)		J011 (FBOX1, Potri.006G068500, Chr6)	
	SD09	SD_RH_sp	Spring—mean annual relative humidity (%)	K016 (WRKY10, Potri.014G155100, Chr14)		
	SD10	SD_RH_sm	Summer—mean annual relative humidity (%)			
	SD11	SD_RH_at	Autumn—mean annual relative humidity (%)			
	SD12	SD_EMT	Extreme minimum temperature over 30 years		J011 (FBOX1, Potri.006G068500, Chr6)	
	SD13	SD_EXT	Extreme maximum temperature over 30 years	L026 (PTH1, Potri.012G130400, Chr12) L034 (IAA1, Potri.014G135300, Chr14)		
Soil variables	Soil01	S_SAND	Subsoil sand fraction (% weight)			
	Soil02	T_GRAVEL	Topsoil gravel content (% volume)			L029 (GH3‐1, Potri.013G151100, Chr13) K008 (WRKY4, Potri.006G224100, Chr6)
	Soil03	T_SILT	Topsoil silt fraction (% weight)			
	Soil04	S_C	Dominant soil type subsoil carbon content (kg C/m^2^)			
	Soil05	S_CEC_CLAY	Cation exchange capacity of the clay fraction in the subsoil (cmol per kg)			
	Soil06	S_CLAY	Subsoil clay fraction (% weight)		L039 (CAM1, Potri.018G111200, Chr18)	
	Soil07	S_GRAVEL	Subsoil gravel content (% volume)	K008 (WRKY4, Potri.006G224100, Chr6)		
	Soil08	T_BULK_DEN	Topsoil bulk density (kg/dm^3^)			

#### 2.4.2 Biotic factors

Population genomic information for *S. musiva* was obtained from a previous study (Sakalidis, Feau, Dhillon, & Hamelin, 2016). Haplotypes for 323 SNPs and representing 59 individuals (distributed in 10 populations) were used to compare the geographical structure of the pathogen with its host tree. The *P. deltoides* dataset was randomly subsampled to create a similar‐sized dataset because the fungal sampling was less extensive than tree sampling. We selected trees from geographical locations that were close or identical to pathogen origin. Concordance between the two datasets was measured using the procrustes analysis implemented in the R vegan package (Oksanen et al., 2016).

### 2.5 Genetic‐environment association analyses

Before analyzing genomic and environmental data associations, we performed a PCA for the environmental variables alone. We then used variance partitioning (Borcard, Legendre, & Drapeau, 1992) to quantify the separated and combined effects of four groups of explanatory variables for *P. deltoides* genomic diversity. The four partitions were (a) soil, (b) geographical, (c) climate mean, and (d) climate standard deviation variables. Adjusted *R* ^2^ was used to measure the importance of each fraction in the variance partitioning. Moreover, associations between these explanatory variables and tree genomic variation were also tested using redundancy analysis (RDA). This analysis was conducted on two different datasets: first, on “pure” individuals presenting Structure *Q*‐values >0.9 and, second, on admixed trees presenting Structure *Q*‐values <0.9. Our objective in this case was to test whether similar genotype–environment associations occur in both “pure” and admixed regions. The idea was to look for specific environmental variables that would explain the lineage differentiation not only in allopatric regions with very different environmental conditions but also in sympatric zones that presented a mix of conditions.

The variance partitioning was performed using the "varpart" function; the PCA and RDA were performed using the "rda" function. The significance of the associations was tested using the "ANOVA" function with a step size of 10,000, resulting in at least 9,999 permutations. These functions were all implemented in the R vegan package (v2.5‐3; Oksanen et al., 2016).

To identify specific SNPs that were associated with distinct environmental variables, we used latent factors mixed models (LFMM) (Frichot, Schoville, Bouchard, & François, 2013) as implemented in the Bioconductor LEA package (v2.6; Frichot & François, 2015) in R. The LFMM method is designed to deal with the lack of neutral dataset and appeared to be best suited for studies that presented a high level of population structure (Lotterhos & Whitlock, 2014; Rellstab, Gugerli, Eckert, Hancock, & Holderegger, 2015). This method uses a hierarchical Bayesian mixed model to detect correlations between environmental and genomic variations while simultaneously inferring population structure. The correction for the putative neutral background structure was performed by introducing *k* latent factors, determined using a first‐step PCA method, into the model. The number of significant latent factors was identified using a Tracy–Widom test. The LFMM analysis was then performed for the previously determined (*k*) number of latent factors and was run for 10 independent runs, each using 10,000 iterations with the first 5,000 iterations discarded as a burn‐in. The independent replicates were then combined using the Fisher–Stouffer method, and the resulting *p* values were adjusted using the genomic inflation factor (λ) and then corrected for multiple testing using a false discovery rate of .05 (Benjamini & Hochberg, 1995). The LFMM analyses were performed using the selection of 29 variables (26 abiotic and three geographic) separately and using the 93 successful SNPs.

### 2.6 Sequence capture on pool approach

In order to compare the genome‐wide variation and differentiation between the different genetic lineages identified with our SNP array, we conducted a sequence capture experiment on pools of trees representative of the different groups. We selected probes from the *P. trichocarpa* genome v3.0 to cover almost completely 1,470 genes potentially involved in stress responses, phenology, and secondary metabolic pathways to build a 5 Mb capture library (SeqCap EZ, Roche Nimblegen). We used four pools of 20 diploid individuals: three were comprised of trees presenting Structure *Q*‐values >0.9 for each of the three Structure groups identified: the subsp. *monilifera*, the northeastern subsp. *deltoides*, and the southern subsp. *deltoides*, and the fourth pool was comprised of trees located in the admixed region between the southern and northeastern *deltoides* subsp. groups (*Q*‐values < 0.9) (Table S1). The DNA concentration of each individual within each pool was adjusted equally to avoid sequencing bias. The four libraries were then paired‐end sequenced (2 × 100 bp) with an Illumina MiSeq sequencer (Illumina). The sequences were aligned on the WV94 *P. deltoides* genome v2 (phytozome.jgi.doe.gov), a clone from Issaquena County, Mississippi, with the CLC Genomics Workbench v.7.5.1 (QIAGEN), keeping only unbroken sequence pairs. We then identified the variants within each pool, and on the combination of the four pools, using the low‐frequency variant detector in the same software suite: freq. threshold = 2%, min. cov. = 80 (individual pools) or 200 (merged pool), and min. qual. = 20. Only the biallelic SNPs that appeared in the individual pools, with a coverage comprised between 150 and 500, and merged pools were kept. We also kept only SNPs included within the 19 chromosomes of the genome assembly by excluding the few SNPs observed in other scaffolds. Observed pool allele frequencies below 2% were brought down to 0% as they were under the detection limit.

We used the SNP allele frequency estimates in each pool to draw allele frequency spectrum (AFS) plots (Gutenkunst, Hernandez, Williamson, & Bustamante, 2009; Sousa & Hey, 2013). This approach portrays the actual state of allelic differences between two populations and allows us to make assumptions on how two groups diverged and, especially, how they have exchanged gene flow over time. We compared our AFS plots presenting the observed allele frequency measured in our sampling with AFS representing different models (isolation, isolation with migration, isolation after migration, and secondary contact; Sousa & Hey, 2013) to determine which divergence model best corresponds to our data. The AFS plot is a bidimensional matrix where each *x_ij_* point corresponds to the number of SNPs found with a specific allele frequency combination in each pair of populations (pools) that are compared. Hence, in the absence of gene flow between the two populations, the frequencies of SNPs found in only one population are different from the SNPs in the other populations as a consequence of the genetic drift; but, in models with gene flow, we should observe many SNPs with similar frequencies in the two populations. Each plot was composed of a 41 × 41 grid that corresponds to the 41 possible allele frequencies in each pool; that is, considering *N* and *M* individuals in each populations, the AFS matrix should be (2*N* + 1) × (2*M* + 1), to allow for derived allele counts of 0. We produced six AFS plots to compare each pair of pools.

The allele frequency measures were also used to calculate the expected heterozygosity (*H*_e_) for each SNP per group. We then obtained the total heterozygosity (*H*_t_) using the mean *H*_e_ per SNP for each group. Using these values, we were able to calculate the differentiation index (*F* _st_) (a) between each pair of pools (including the SNPs that were monomorphic for the pairs of pools compared) and (b) each single pool v. the three others, to draw Manhattan‐like plots (presenting the *F* _st_ distribution rather than *p* values on the *y*‐axis). We used this approach to visualize the divergence impact at the genomic scale by identifying chromosomic regions that presented higher *F* _st_. We also used them to visualize the SNPs previously identified by the FDist2 and LFMM analyses as being potentially under selection.

## 3 RESULTS

### 3.1 Data preparation

We retained genotype information for 946 trees representing 251 different localities after the removal of putative hybrids, clones, and individuals missing more than 10% of genotypes (Table S1). Of the 102 nuclear SNPs tested, 93 were successful. From the cpSNP assay design, we obtained five successful SNPs but only three of these were informative (polymorphic and in linkage disequilibrium) and thus retained. The combination of chloroplastic information resulted in only three distinct haplotypes, so no further analysis was performed. Their geographical distribution was highly structured (Figure 1b) and comparable to nuclear data.

### 3.2 Population structure

The Structure analysis based on the SNPs described above indicated that a three group cluster was the best configuration (according the Delta *K* statistic). The Structure partition was also supported by the PCA analysis (Figure S2a), where the first axis (16.0%) differentiates trees from the northwestern region from the rest. It corresponds to the distinction between the *monilifera* and *deltoides* subspecies. The second principal component axis (4.7%) separated trees from the northeastern region from those of the southcentral region. These two groups correspond to the same *deltoides* subspecies. The Structure analysis conducted using alternative parameters and the PCA performed on several random subsamples led to the unequivocal identification of the same three groups (Figure S3). In addition, plotting the Structure *Q*‐values showed that both the 0.9 probability intervals inferred for individuals having *Q*‐values >0.9 or *Q*‐values >0.667 do not overlap (Figure S4). The PCA of environmental variables (climate mean and standard deviation and soil) also showed the same clustering pattern (Figure S2b). Neither the cpDNA nor ncDNA population structures fitted the delineation of the ocean watersheds (Figure 1a, b).

The Structure and PCA results also showed that the three major groups integrated smoothly at their borders. Most of the admixed individuals had a genomic background from the two northern groups (*monilifera* subsp. and northeastern *deltoides* subsp. groups). The AMOVA analysis showed a very strong differentiation between the three clusters (Table 2), where the most differentiated groups were the southern *deltoides* subsp. and *monilifera* subsp. (*F*_CT_ = 0.284). Within clusters (Table 3), the differentiation among populations was much lower (*F* _sc_ = 0.041). There was no significant difference between each population differentiation (*F* **_st_**) and inbreeding values calculated for each of the three clusters. Genomic diversity appeared significantly higher in the subsp. *deltoides* (northeastern and southern groups) than in the *monilifera* subsp., a tendency that was confirmed at the genome scale (see below). The Hardy–Weinberg equilibrium tests detected a slight deficit in heterozygotes in the two northern clusters, that is, the *monilifera* subsp. (*F*_is_ = 0.021) and the northeastern *deltoides* subsp. cluster (*F*_is_ = 0.030), as well as in the admixed group (*F*_is_ = 0.081).

**Table 2:** Differentiation indices between the three lineages identified by the Structure analysis using the SNP assays (AMOVA *F* _ct_, upper half). Pairwise *F* _st_ calculated between the different pools using the 54,712 SNPs from the sequence capture method (lower half)

*F* _st_/*F* _ct_		Subsp. *deltoides*			Subsp. *monilifera*
		Northeast	Admixed	South	West
Subsp. *deltoides*	Northeast	.	–	0.188	0.241
	Admixed	0.035	.		–
	South	0.050	0.030	.	0.284
Subsp. *monilifera*	West	0.063	0.049	0.061	.

**Table 3:** Differentiation and diversity indices for the entire sample and the three Structure clusters when classifying individuals in one of the groups using a Structure *Q*‐value >0.667 and considering only populations with three individuals or more (corrected for the sampling size)

Group	*n*	*n*. pop	*F* _st_	*H*_o_	*H*_s_	*H*_T_
Northeastern subsp. *deltoides*	84	10	0.055	0.312^1^	0.308^2^	0.3183^3^
Southern subsp. *deltoides*	54	9	0.047	0.309^1^	0.295^2^	0.3021^3^
Subsp. *monilifera*	432	41	0.038	0.261	0.259	0.270
Total	779	90	0.1466	0.284	0.283	0.333

Note

^1,2,3^No significant difference between values.

### 3.3 Chloroplast DNA capture

When we compared the population structure obtained from the analysis of nuclear DNA with the cpDNA haplotypes, we observed individuals that presented a mixed composition of cpDNA and nuclear DNA, that is, a cpDNA typical of one lineage or subspecies in combination with a genetic cluster (based on nuclear DNA) associated to another lineage or subspecies. This type of pattern is the consequence of cpDNA capture (i.e., the replacement of cpDNA from one lineage by that of another). More specifically, we observed 37 trees presenting a *deltoides* subsp. nuclear makeup with a cpDNA typical of the *monilifera* subsp. versus eight trees for the opposite relation (Figure 1b).

### 3.4 Outlier analysis

The outlier test permitted the identification of several loci that presented a significant amount of differentiation between the analyzed genetic clusters. Only loci that appeared significant in each of the four configurations tested were retained, that is, for the two cluster assignments (using a Structure *Q*‐value threshold of 0.667 and of 0.9) and using a minimum number of three or five individuals per population (Table 4). We concluded that a specific lineage was responsible for the differentiation observed in pairwise outlier analyses (one lineage vs. another one, for a total of four pairwise analyses), when the same locus appeared significant in the two analyses in which one of the lineage was included.

**Table 4:** Outlier loci identified by the FDist2 analysis for a three cluster configuration and for each pair of lineages

3 clusters	Northeast vs. south	West vs. south	West vs. northeast
J007 (DIR2, Potri.003G134600, Chr3)	*J007 (DIR2, Potri.003G134600, Chr3)*	*J007 (DIR2, Potri.003G134600, Chr3)*	**J026 (WRKY1, Potri.014G096200, Chr14)**
J026 (WRKY1, Potri.014G096200, Chr14)	K003 (Potri.003G134600, Chr3)	**J026 (WRKY1, Potri.014G096200, Chr14)**	K010 (EXPANS4, Potri.009G169500, Chr9)
K008 (WRKY4, Potri.006G224100, Chr6)		J039 (FBOX2, Potri.005G170000, Chr5)	**L031 (HDZIP1, Potri.014G075200, Chr14)**
L011 (GT1, Potri.002G200200, Chr2)		K008 (WRKY4, Potri.006G224100, Chr6)	
L031 (HDZIP1, Potri.014G075200, Chr14)		**L031 (HDZIP1, Potri.014G075200, Chr14)**	

Note

Bold indicates loci that are associated with the differentiation of individuals from the western cluster (subsp. *monilifera*), and italics indicates loci were associated with the differentiation of the southern cluster (subsp. *deltoides*). Other loci are those that are significant in the three clusters or pairwise analysis. In parenthesis, putative gene name, *Populus trichocarpa* gene name, and chromosome number on which the locus is found.

### 3.5 Genetic‐environment associations

#### 3.5.1 Abiotic variables

The variance partitioning analysis showed that abiotic variables explained 20.9% of the genomic variation (Figure S5). Most of this explained variation was associated with the interactions of the different environmental variables (18.5%). Moreover, the analysis showed that the stand‐alone contributions from geography, soil, standard deviation, and mean climate variables were responsible, respectively, for 0.26%, 0.34%, 1.31%, and 0.50% of the variation.

**Figure 2:**
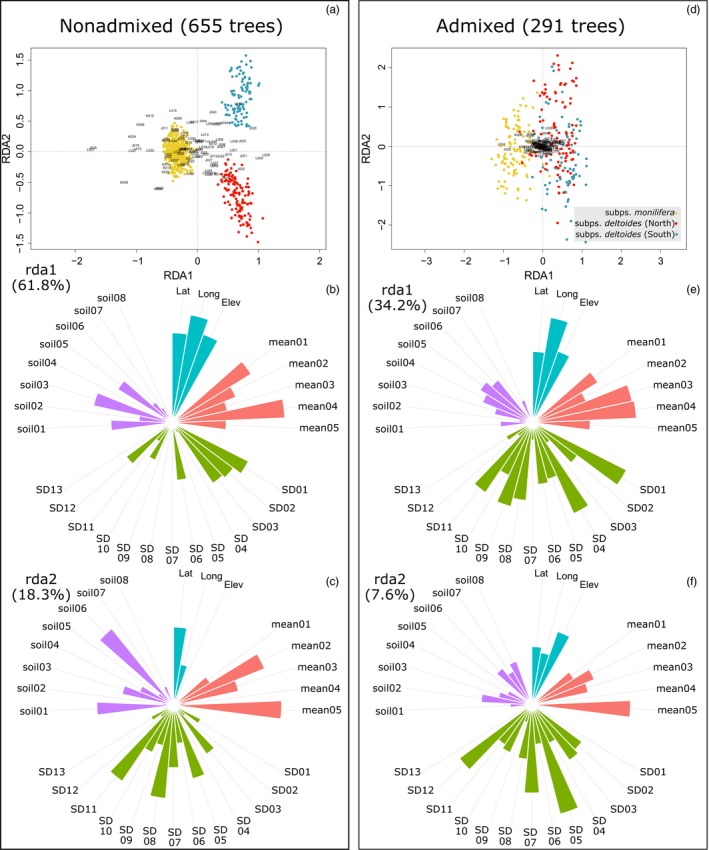
Redundancy analysis (RDA) ordination plots from the redundancy analysis representing (a) all nonadmixed trees (*Q*‐values > 0.9) and (d) admixed trees (*Q*‐values < 0.9). The colors of the points correspond to the Structure assignment results (using the maximal *Q*‐value to determine the group) and the marker names are in black. (b, c, e, f) Polar plots represent the absolute score values of the different environmental variables used in the RDA for the first and the second axis of the analysis performed on nonadmixed and admixed trees. Different colors correspond to the different categories of environmental variables. The names of the corresponding variables are in Table 1

The RDA indicated that both datasets (“pure” and admixed trees) showed similar patterns (Figure 2) and these were congruent with PCA performed on genomic and abiotic data separately (Figure S2b). The first axis differentiated the two subspecies (*monilifera* and *deltoides*), while the second axis separated the northern and southern groups of the *deltoides* subspecies. For “pure” and admixed datasets, 28.6% and 18.1%, respectively, of the total genomic variance were constrained by our set of noncorrelated variables. Similarly, the two first axes explained about twice the variation in the “pure” dataset compared with the admixed one (Figure 2). The first 16 and 11 axes for “pure” and admixed datasets, respectively, were significant (*p* < .05). All climate variables were highly significant (*p* < .001) for the “pure" dataset, while the only significant soil variables were S_C (subsoil carbon content, *p* = .021), S_CEC (cation exchange capacity of the clay faction in the subsoil, *p* = .002), and S_GRAVEL (subsoil % gravel, *p* = .013). Similarly, all climate mean variables were highly significant for the admixed analysis and about half of the soil and climate standard deviation variables had a *p* value <.05 (data not shown).

The preliminary PCA analysis and the Tracy–Widom test indicated that five latent factors should be used for the LFMM analyses. Significant associations were identified between 16 distinct SNPs and environmental variables tested. Details are presented in Table 1 and in Figure S6.

#### 3.5.2 Biotic variable: Host–pathogen association

The assessment of the congruence between the population genomic structure of *P. deltoides* and its native fungal pathogen (*S. musiva*) revealed a strong correlation (.800, *p* = .001). Although pathogen sampling was sparser than for its host tree, the visual comparison of the two Bayesian clustering results (Figure 1a,c) also strongly supported a correlation between the two geographic patterns.

### 3.6 Genomic variation detected between the different lineages at the scale of the whole genome

We identified 54,712 SNPs from our sequence capture procedure conducted on the four pools (4 × 20 individuals). From these, 24,526 were polymorphic in each pool and the southern *deltoides* subsp. showed the highest number of endemic SNPs (i.e., found to be polymorphic only in this group, Table 5). The mean *H*_T_ comparison between each group and the pairwise *F* _st_ comparisons mostly fitted the diversity and divergence measured with the SNP array, where the *monilifera* subsp. appeared to be the least diverse (Table 5) and the most differentiated group with higher *F* _st_ peaks (Table 2 and Figure S7).

**Table 5:** Differentiation and diversity indices obtained from the sequence capture approach conducted on pools of trees from the three genetic groups identified by Structure analysis and one admixed group (between the two subsp. *deltoides* groups)

DNA pools	*n*	*F* _st_ (one group vs. the three others)	Polymorphic loci	Endemic SNP^a^	*H*_T_
Northeastern subsp. *deltoides*	20	0.038	22,225 (40.6%)	3,204 (1,369)	0.164
Southern subsp. *deltoides*	20	0.034	30,100 (55.0%)	7,236 (3,725)	0.175
Admixed subsp. *deltoides*	20	0.020	24,813 (45.4%)	3,187	0.188
Subsp. *monilifera*	20	0.050	21,804 (39.9%)	3,465 (2,409)	0.159
Three groups (without the admixed)	60	0.086^b^	51,525 (94.2%)	12,808^c^	0.191
Total	80	0.009^b^	54,712	–	0.194

^a^ Number obtained when excluding the admixed pool from the calculation (In parentheses: number obtained including the admixed pool in the calculation).

^b^ *F* _st_ measured between the four and three groups.

^c^ Monomorphic in the admixed region.

**Figure 3:**
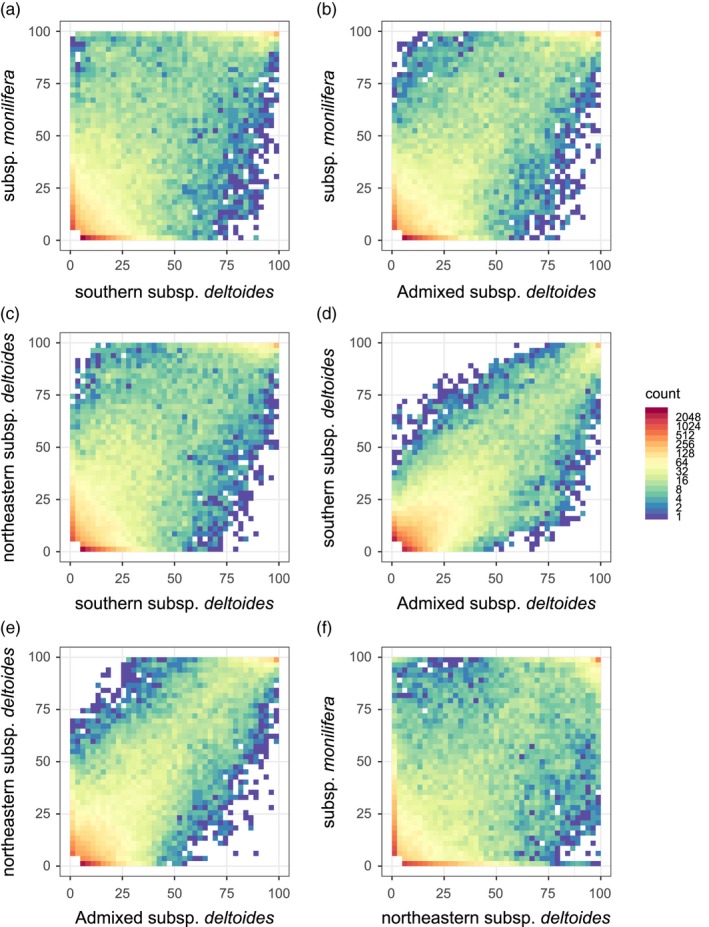
Allele frequency spectrum (AFS) plots presenting the number of SNPs showing a specific allele frequency in each pair of groups. For visual purposes, the counts for allele frequencies smaller or equal to 5% in both compared groups were removed (white square in the lower left corner). Graphics (a)–(f) correspond to each of the six possible pairs of comparison

When interpreting the results from the AFS plots in light of the review by Sousa and Hey (2013), we detected a pattern that fits an isolation‐with‐migration model of divergence for the comparison between the southern *deltoides* subsp. and *monilifera* subsp. (Figure 3a). Nevertheless, it should be noted that such a pattern can be very difficult to distinguish from isolation after migration or a pattern of secondary contact (Sousa & Hey, 2013). A very similar pattern is observed between the subsp. *deltoides* admixed group and the *monilifera* subsp.; that is, an excess of alleles of high frequencies in the *monilifera* subsp. is observed at low frequencies in the *deltoides* subsp. (Figure 3b). A comparable, but weaker, signal is also observed when comparing the two *deltoides* subsp. groups (Figure 3c). The relation between the *deltoides* subsp. admixed group and the southern or northeastern *deltoides* subsp. groups showed low differentiation as the new alleles (observed at low frequencies) are often shared between the groups (Figure 3d,e). At the opposite end, the comparison of the northeastern *deltoides* subsp. and *monilifera* subsp. groups suggests limited gene flow and higher differentiation since the emergence of new alleles was restricted to one group most of the time and alleles with high frequencies appear quite unbalanced (Figure 3f). Also, differences between effective population size (*N*_e_) might likely result in a similar trend. The Manhattan‐like plots (Figure S7) showed that SNPs with high *F* _st_ values often clustered in specific genomic regions. This strongly suggests that these regions could potentially be under selection, although the pool approach prevented us from testing this using traditional gene association method. The *monilifera* subsp. comparison showed higher *F* _st_ peaks and, as expected, the admixed comparison presented weaker peaks.

## 4 DISCUSSION

We detected a strong geographical structure consisting in three genetic lineages for *P. deltoides*. One of these fits the taxonomic classification of the subspecies *monilifera*, and the two others match the subsp. *deltoides*; one found in the south and the other in the northernmost portion of its range. These three groups were each associated with specific abiotic variations. We also detected a coevolution signal of *P. deltoides* and an associated pathogen, *S. musiva*. In addition, we identified several SNPs that could be associated with environmental conditions and adaptations. These results were consolidated by the sequence capture approach using pools of trees representative of each lineage. This method allowed us to confirm a scenario of divergence with directional gene flow between the two northern groups (west and east) and the southern group.

The use of SNPs developed from candidate genes allowed us to obtain a general portrait of the genetic diversity within *P. deltoides* as well as to look for DNA polymorphisms possibly associated with environmental variation. However, the global diversity measured with this SNP set was likely lower than if we would have used noncoding regions (e.g., microsatellites) (Morin, Luikart, Wayne, & The SNP Workshop Group, 2004). This lack of fine resolution can probably explain why we failed to detect a substructure within the three genetic groups that would fit any specific regions or drainage areas (data not shown). Regardless, this ensemble of SNPs allowed us to delineate genetic clusters in order to understand gene flow movement at a broader geographical and genomic scale based on a sequence capture on pool approach.

The distribution of cpDNA diversity compared with the ocean watershed boundaries showed that river flow contributes to the generation of novel assemblages of trees presenting a cpDNA from one lineage and ncDNA genetic makeup from another lineage. However, this gene flow between the different lineages appears uneven; indeed, the populations located upstream seem to receive less than they give. Interestingly, this gene flow movement is parallel to the change in climatic conditions; that is, the alleles come from regions having climates that correspond to the projected future climates of downstream populations. Globally, this highlights the importance of considering both within‐species differentiation and connectivity between populations in the evaluation of species vulnerability to global change.

### 4.1 Higher structuring than expected but this fits with environmental factors

Among the three subspecies identified*,* two, subsp. m*onilifera* and subsp. *deltoides*, have been delineated using our cpDNA and ncDNA markers. The absence of the *wislizeni* subsp. was not surprising because our sampling did not cover much of the southwestern region. What was most unexpected was the identification of a third group in the northeastern part of *P. deltoides’* natural range in southern Canada. The delineation of this third lineage was detected by the three clustering analyses that we used (Structure, PCA, and RDA analyses) and by the sequence capture approach. Precipitation and winter humidity appeared to be the most important climatic drivers of differentiation between *monilifera* and *deltoides* subsp. (Figure 2). This adaptation to drought is coherent with the smaller leaves found in *monilifera* subsp. (Maini, 1968). Indeed, smaller leaves would favor low epidermal transpiration (Parkhurst & Loucks, 1972; Van Splunder, Voesenek, Coops, De Vries, & Blom, 1996). Such leaf morphological differentiation, at least partially related to climate, has also been previously observed within *Populus nigra* (Dewoody et al., 2015; Van Splunder et al., 1996). Overall, the precipitation regime was associated with the differentiation between the *monilifera* and *deltoides* subsp. and latitude‐dependent variables explained the divergence within the *deltoides* subsp., where the most important climatic variables were mean annual temperature and snowfall (Figure 2c,f). This latitudinal gradient could be associated with bud phenology selection as proposed by Evans et al. (2016) for *P. angustifolia*. Interestingly, photoperiod was reported to be an important factor in explaining the trade‐off between freezing tolerance and growth in a greenhouse experiment conducted using several members of the North American Salicaceae family (Savage & Cavender‐Bares, 2013).

Three different varieties of the *deltoides* subsp. (southern, central, and northern) were proposed in the past based on minor morphological differences (Haverbeke, 1990). These taxa were not officially recognized in subsequent taxonomical revisions. However, it is possible that our northeastern group coincides with the previously defined northern ecotype of *P. deltoides* and effectively presents morphological differences that would indicate environmental adaptation. In this same region, as for *P. deltoides*, a similar genetic break was observed for a yeast species and was interpreted as an adaptation signal for the temperate forest ecosystem (Leducq et al., 2014). An analogous genetic pattern was also observed within boreal *P. balsamifera* populations (Keller, Olson, Silim, Schroeder, & Tiffin, 2010; Meirmans et al., 2017). However, given that it did not coincide with an environmental gradient in *P. balsamifera*, demographic effects rather than adaptation to climate were proposed to explain this sharp pattern. Such effects could also have contributed to the genetic differentiation of the northeastern group of *P. deltoides* and thus cannot be ruled out from our interpretation, even if both abiotic and biotic factors were correlated with this clustering. Indeed, *P*. *deltoides* migration in the northeastern region is relatively recent since it was glaciated during the last ice age (Dyke et al., 2002). Hence, an effect of vicariance in a distinct refugium or a differentiation during the northward migration of the species (i.e., allele surfing; Excoffier & Ray, 2008) could have produced a similar genetic pattern. No *Populus* pollen data are available that could help understand the postglacial colonization of this species (P.J.H. Richard, pers. comm). That being said, we cannot exclude the possibility that the *deltoides* subspecies differentiation observed could have resulted from an isolation by distance effect, although the two complementary Structure and PCA analyses also pointed toward a three clusters configuration (Figure S3). A strong population structure was also observed in previous genomic diversity studies of *P. deltoides* in the U.S Southeastern region; but the groups detected were found in areas not covered by our sampling (Fahrenkrog, Neves, Resende, Dervinis, et al., 2017; Fahrenkrog, Neves, Resende, Vasquez, et al., 2017), so comparisons with our results cannot be made.

The three different lineages identified were treated as different taxa under allopatric and sympatric speciation scenarios. The comparison of RDA results from “pure” and admixed individuals showed which environmental variables appear to be the most critical in explaining tree distribution, that is, not only in separate and highly different ecoregions but also in areas presenting a blend of habitats. Hence, we considered that a variable would be tightly linked to the lineage differentiation if it appeared important in both sympatric and in allopatric conditions, respectively represented by admixed and nonadmixed (or "pure") individuals. Only variables from the “climatic means” category appeared to satisfy this condition. Soil variables and climate standard deviations, which represent climate variability, had quite different patterns for “pure” and admixed individuals (Figure 2). According to our data, none of the soil characteristics tested appeared to play an evident role in explaining the distribution of the different lineages. This is not to say that edaphic characteristics were not significant at all, but our analysis failed to identify specifically which would be more predominant. It is also possible that smaller effects from edaphic or climate variability were blurred because of the collinearity inherent to the use of large climate, edaphic, and soil datasets. Climate datasets are generated using geographical data to allow interpolation between weather stations (Wang, Hamann, Spittlehouse, & Aitken, 2006; Wang et al., 2012), and climate data are used to generate soil maps (Mansuy et al., 2014; Wieder, Boehnert, & Bonan, 2014; Wieder, Boehnert, Bonan, & Langseth, 2014). This effect can also explain the fact that most of the genomic variability was related to the interactions between the different types of abiotic variables (Figure S5). Hence, it is difficult to separate the different single effects to compare the relative importance of each category in explaining genomic diversity.

The calculation of standard deviation (*SD*) indices for climate data was conducted as an attempt to capture an adaptive plasticity signal (Nicotra et al., 2010). We postulated that the capacity of *P. deltoides*, a riparian species, to deal with variable conditions could be related to its genomic variability because it is tightly associated with a regime of frequent disturbances. However, the interpretation of our results is not straightforward. When comparing the RDA obtained with “pure” and admixed individuals, patterns of climatic *SD* variables were dissimilar. This gives less weight to these variables in effectively explaining genomic variability. An alternative explanation of these results could be that the relationship between the standard deviation climatic variables and genetic diversity is nonlinear (Arnold, Kruuk, & Nicotra, 2019) and could not, therefore, be detected by our methods. Nonetheless, we remain convinced that evolving plasticity likely plays a key role in adaptations to changing environments (Kelly, 2019) and we should continue to work at trying to better understand this phenomenon.

#### 4.1.1 Biotic factor: strong congruence between the fungus pathogen and its host

We found a very high congruence between the genomic diversity found in *P. deltoides* and in one of its associated pathogens, *S.* *musiva.* As in its host tree, three distinct lineages of the pathogen have been identified (Sakalidis et al., 2016) (Figure 1c). This strong phylogeographic co‐signal may indicate coevolution between the tree and the fungus. In a host–pathogen association, coevolution can lead to an incessant evolutionary arms race between the pathogen and its host (Clay & Kover, 1996). Given that different arms races appear to occur in the three different lineages of the *P. deltoides*/*S. musiva* system, we can postulate that different weapons and protection mechanisms may have independently evolved in each of the three geographic regions. Such intraspecific differentiation may represent an advantage for *P. deltoides*, but not for its pathogen, because gene flow appeared to be much more restricted in *S. musiva* (Feau, Hamelin, Vandecasteele, Stanosz, & Bernier, 2005) while *Populus* seeds can travel by stream and pollen is dispersed by wind. The arrival of new *Populus* genotypes presenting new protection mechanisms may counterbalance the faster adaptive capacity of the fungus that can complete its life cycle within 1 year (Thompson, 1941) while *P. deltoides* reaches maturity between 5 and 10 years (Haverbeke, 1990). Nonetheless, the adaptive significance of this congruent pattern between a host tree and its fungal pathogen remains to be tested experimentally.

Few studies have shown evidence of shared population structure between a natural host and its pathogen even if host shift appeared to be important in driving diversification of host–pathogen systems (Floate, Godbout, Lau, Isabel, & Whitham, 2016; de Vienne et al., 2013). The main reason for this may be that most pathogen systems studied concerned crop species or host/pathogens of exotic origin that did not reflect naturally occurring processes (see examples in McDonald & Linde, 2002). Recently, evidence of coevolution between postglacial lineages of an annual plant and its associated fungal pathogen has been detected where the local plant lineages appear to be better adapted to the local pathogen (Feurtey et al., 2016). Such results highlight the utility of using within‐species variability to better understand mechanisms of resistance for plants and of dispersion and virulence for pathogens.

### 4.2 What about the genes?

To study adaptation at both the species and genomic scales, we first used a low (but selected) number of SNPs from a large number of individuals that covered the entire range of *P. deltoides*. This allowed us to design a sequence capture experiment using a small (but selected) number of trees representative of the different lineages we detected in the entire sampling. The combination of these two approaches provided better support for some SNPs identified using the FDist2 or LFMM analyses, identified more genes potentially under selection, and, at the genome scale, confirmed and detailed the differentiation pattern we observed with the small genotyping SNP assay.

From the 18 SNPs previously identified as potentially under selection by the FDist2 and LFMM analyses, 14 were present in the sequence capture dataset and identified in the Manhattan‐like plots (Figure S7). Congruence between the individual (FDist2 and LFMM) and pool (sequence capture) approaches appeared especially high considering the divergence at the whole sampling scale or from the *monilifera* subsp. (Figure S7a,b). Although these SNPs were not all located in chromosomic regions presenting high *F* _st_values, it is interesting to note that four of the five SNPs identified by the FDist2 analyses on three clusters (J007, J026, K008 and L031) were all found in genomic regions with high *F* _st_ values between the three pools (Figure S7a). Less congruence was observed when considering differentiation between the northeastern and southern *deltoides* subsp. groups, possibly because these two lineages were less divergent than the *monilifera* subsp., so their specific signal was diluted.

Of the six SNPs identified to be putatively under selection in the *P. balsamifera* study (Meirmans et al., 2017), one was found in the same gene (but at a different position) as for *P. deltoides* (identified as J026 in the present study). The corresponding gene, WRKY‐1, could be associated with drought adaptation in both species. A similar interpretation could be proposed for the three SNPs found in the genomic region presenting high differentiation (J007, K008 and L031, Figure S7b) that were associated with continentality (K008) and precipitation (J007 and L031) by the LFMM analysis (Figure S6). The L031 SNP, in particular, was found in a region on chromosome 14 that covered around 6 Mb and that presented *F* _st_ values that varied between 0.7 and 0.8 (divergence measurement between *monilifera* and the other groups; Figure S7b). This region corresponds to the feature podel.14G075800 (WV94 *P. deltoides* genome v2, phytozome.jgi.doe.gov) that is identified as a coding region. The J026 and K008 SNPs are both located in a WRKY transcription factor gene (WRKY1 and WRKY4, respectively) while L031 is located in a HDZIP transcription factor gene. The WRKY genes identified in the present study were not the same previously identified to be involved in resistance to *Melampsora* rust fungi (Azaiez et al., 2009). Nonetheless, WRKY transcription factor family members are thought to be involved in biotic and abiotic stress responses (Pandey & Somssich, 2009). The genes identified here were not found to be under selection in other association studies that have focused on *P. deltoides* (Fahrenkrog, Neves, Resende, Dervinis, et al., 2017) or *P. trichocarpa* (Chhetri et al., 2019; Evans et al., 2014; Street et al., 2006). The absence of correspondence is not surprising since the association of the same genes to environmental variables is not common even when the genes tested, sampling design, and analyses are conducted in the same way (Cullingham, Cooke, & Coltman, 2014, but see a counterexample in Fahrenkrog, Neves, Resende, Dervinis, et al., 2017). Further functional studies are necessary to understand how these genes play a role in species adaption to different environments.

Criticism has been voiced concerning biological narratives extracted from literature and data mining information for genes identified by genome scan analyses (Pavlidis, Jensen, Stephan, & Stamatakis, 2012). Such analyses are well known to be prone to detecting false positives (Lotterhos & Whitlock, 2014; de Villemereuil, Frichot, Bazin, François, & Gaggiotti, 2014). Hence, we consider that our approach, which combines traditional SNP genotyping with a sequence capture method, may help make better informed decisions before proceeding to a formal validation stage. Although our approach does not overcome all the cautionary statements of outlier‐type or genetic association methods, it does provide additional indication of the likelihood that the identified SNPs or regions are potentially under selection for less studied species like *P. deltoides*. The consecutive experiments that would formally validate the adaptive role of a SNP or a gene are often more complex than the gene identification experiments that precede them. For example, these types of validation tests may use, in addition to genomics, genetic transformation (Fernandez i Marti & Dodd, 2018), biochemical procedures (Mageroy et al., 2015), and/or studies in greenhouses or common gardens (Avia, Kärkkäinen, Lagercrantz, & Savolainen, 2014) that may be challenging and time‐consuming, especially when conducted on long‐lived species like trees. Hence, we propose that this type of combined methodology could be useful for selecting the most promising SNPs and genes potentially involved in adaptation to different habitats within a same species.

### 4.3 Enrichment through river flow

Given that *P. deltoides* is associated with riparian habitats, we expected that its genomic diversity might have been connected to the delineation of the drainage basins which was the case for *Populus fremontii* (Evans et al., 2015), a sister species found in the American Southwest. At the largest scale, the ocean drainage basins did not coincide with genomic lineage borders (Figure 1a). However, when the geographic distributions of both the ncDNA and cpDNA genomic variants are superimposed, we observe that rivers appear to contribute to gene exchanges between the different lineages (Figure 1b). The disequilibrium of maternally inherited chloroplast genome capture events between the subsp. *monilifera* and subsp. *deltoides* lineages is a probable consequence of river flow direction differences. Since *P. deltoides* seeds can also be dispersed by wind (Braatne et al., 1996), it is worth noting that prevailing winds blow in the same direction as the river flow in the two regions concerned (Barry & Chorley, 2003). Such organelle genome capture has been previously observed between and within forest species as a consequence of postglacial migrations (Du, Petit, & Liu, 2009; Gérardi, Jaramillo‐Correa, Beaulieu, & Bousquet, 2010; Godbout, Yeh, & Bousquet, 2012). Since cpDNA is dispersed by seeds, which is less effective than pollen dispersal, the cpDNA capture events we detected may be ancient (Godbout & Bousquet, 2014; Petit & Excoffier, 2009). Given that our analyses showed that each of the lineages was associated with distinct environmental features, we can postulate that some lineages may benefit from nonlocal intraspecific variation (i.e., novel alleles), although we did not test for adaptive introgression.

Such unbalanced gene flow between the different lineages could be an example of “unassisted” migration within a tree species. Indeed, gene flow direction in our case is in line with future climate predictions in the continental southern USA. In fact, the effect of climate change has already been observed in the region since the “100th Meridian divide,” which historically separated arid western USA from the humid east, was found to have actually moved toward the east (Seager et al., 2018). Hence, the southern *deltoides* subsp. may benefit from the arrival of new alleles from the western *monilifera* subsp. that would have been naturally selected to be adapted to a drier climate. Similarly, the northeastern *deltoides* subsp. may benefit from the input of new alleles coming from the southern lineages that could exhibit new characteristics that are and will potentially fit the hotter conditions of the projected future climate. Such a phenomenon has recently been documented for populations of an annual wildflower found at the edge of its northern range, where descendants having one parent coming from a warmer southern climate showed higher fitness in warmer conditions than the local plants (Bontrager & Angert, 2019). At this time, we can only hypothesize that adaptive introgression may occur between the different *P. deltoides* groups, but it would certainly be interesting to test this effect on fitness for this riparian tree.

## 5 CONCLUSIONS

With global change, riparian ecosystem functioning, which largely depends on foundation species like *P. deltoides*, is becoming more fragile. Although it remains challenging to translate results from a genomic study to the scale of conservation practices (Shafer et al., 2015), our results for *P. deltoides* can help boost the use of evolutionary processes in forestry management (Lefèvre et al., 2013). Understanding how the genetic diversity of *P. deltoides* is distributed across diverse habitats offers a simple way to assess its vulnerability at the scale of the species range. Our data showed that natural mechanisms are actually in place that may favor the adaptive capacity of the southern and northeastern populations because they can benefit from the input of “preadapted” alleles from adjacent lineages. Although the translation of such gene exchanges in increased fitness for *P. deltoides* remains to be demonstrated, this idea is consistent with the Hamilton and Miller (2016) proposal to take advantage of inter‐species genetic exchanges in management practices in the context of climate change. Hence, for these regions, we recommend soft management measures that, for example, would promote the establishment and regeneration of the species along rivers. In comparison, more drastic actions may be needed to ensure the resilience of the *monilifera* subsp. in the western U.S. and Canada. Our results showed a lower genetic diversity and less gene exchange with other lineages. Moreover, these regions are predicted to be severely impacted by climate change with an increase in aridity (Cook, Woodhouse, Eakin, Meko, & Stahle, 2004). Hence, planting and habitat protection could be needed, as well as the development of breeding programs to select genotypes most likely to survive. In this last case, genetic diversity found in each lineage, as well as in the admixed regions, should receive special attention as they constitute "in situ" reserves of the genetic diversity associated with specific adaptation.

## CONFLICT OF INTEREST

None declared.

## Data Availability

Data for this study are available as Supporting Information.
