# Supplementary material for: Going with the flow: Intraspecific variation may act as a natural ally to counterbalance the impacts of global change for the riparian species Populus deltoides
Source: Evol Appl. 2019 Sep 20;13(1):176–94. doi: 10.1111/eva.12854 (PMC6935597; doi:10.1111/eva.12854)
Supplement: Supplementary file 1 [file EVA-13-176-s001.docx]

## Supplementary materials

**Figure S1** Approximate range distribution of the three subspecies identified for *Populus deltoides* (based on Eckenwalder, 1977)

**
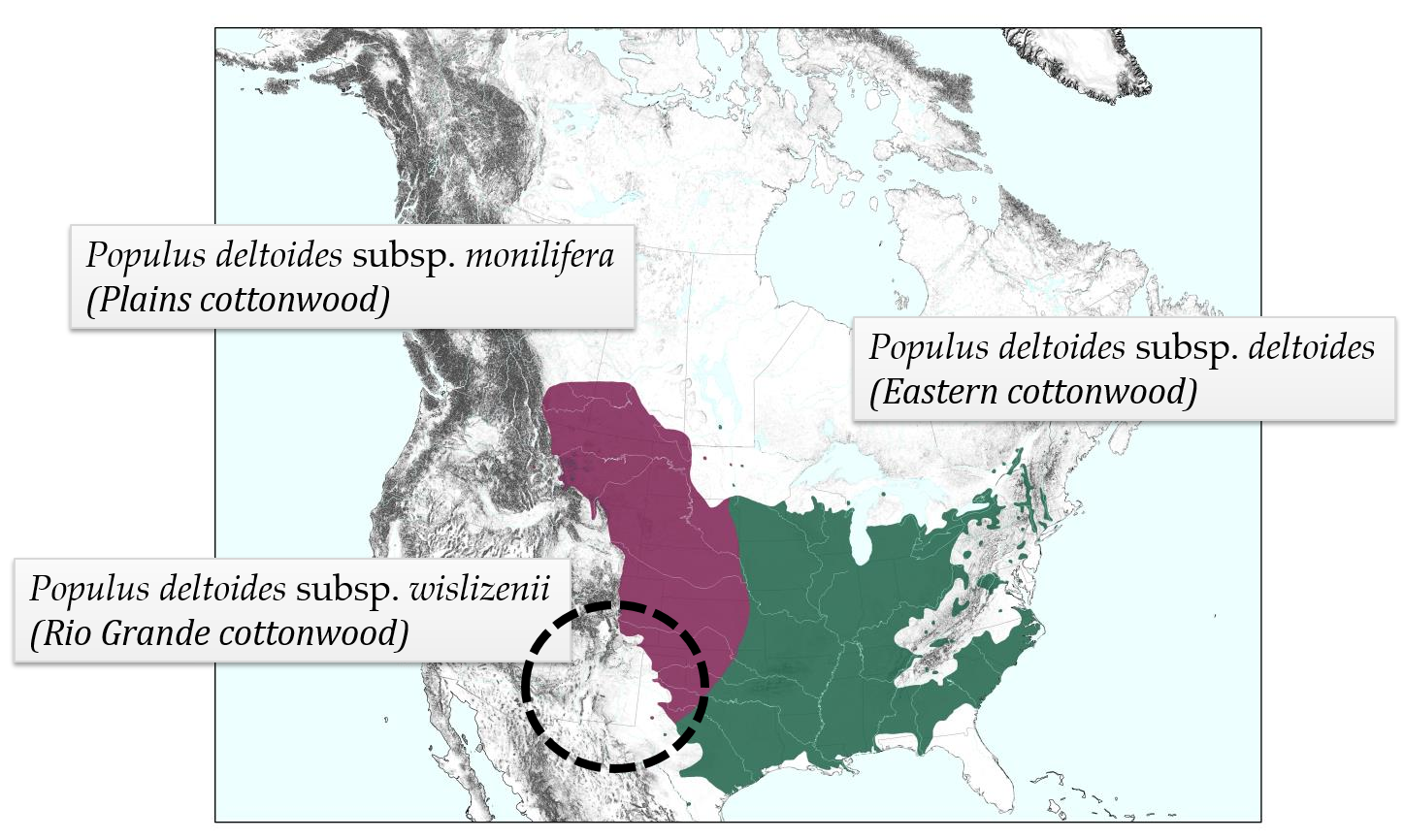
**

**Figure S2** PCA analysis over all a) genetic markers and b) environmental variables. Dots of different colours correspond to the different lineages identified by Structure.
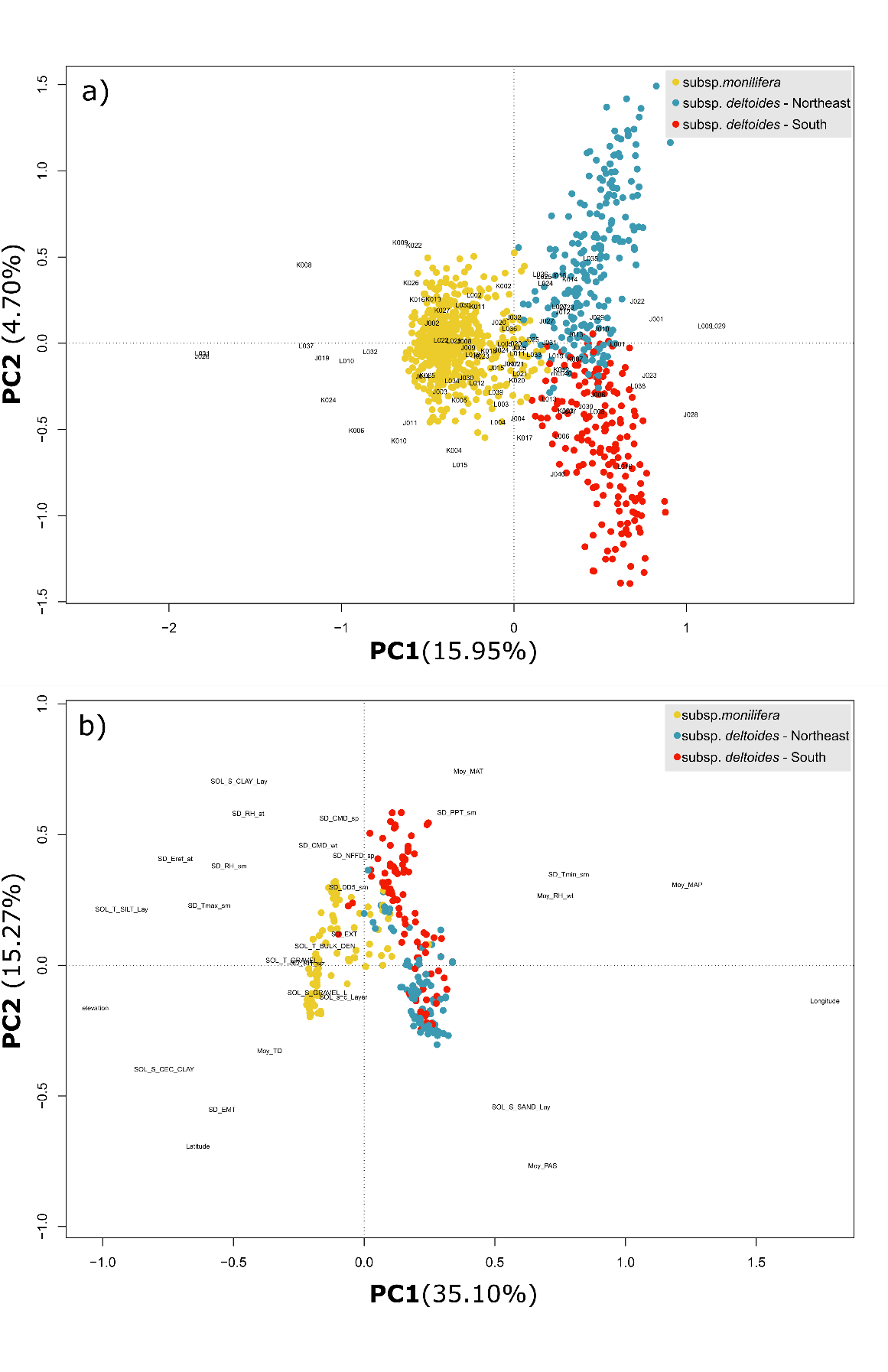


**Figure S3** a) Plot showing Evanno's ΔK with one peak corresponding to *K* = 3 (obtained from the Structure analysis performed using the parameters described in Wang (2017). b) Ordination showing the results from one of the PCA analysis conducted on a random subsample (*n* = dots of different colours correspond to the different lineages identified by Structure.

**
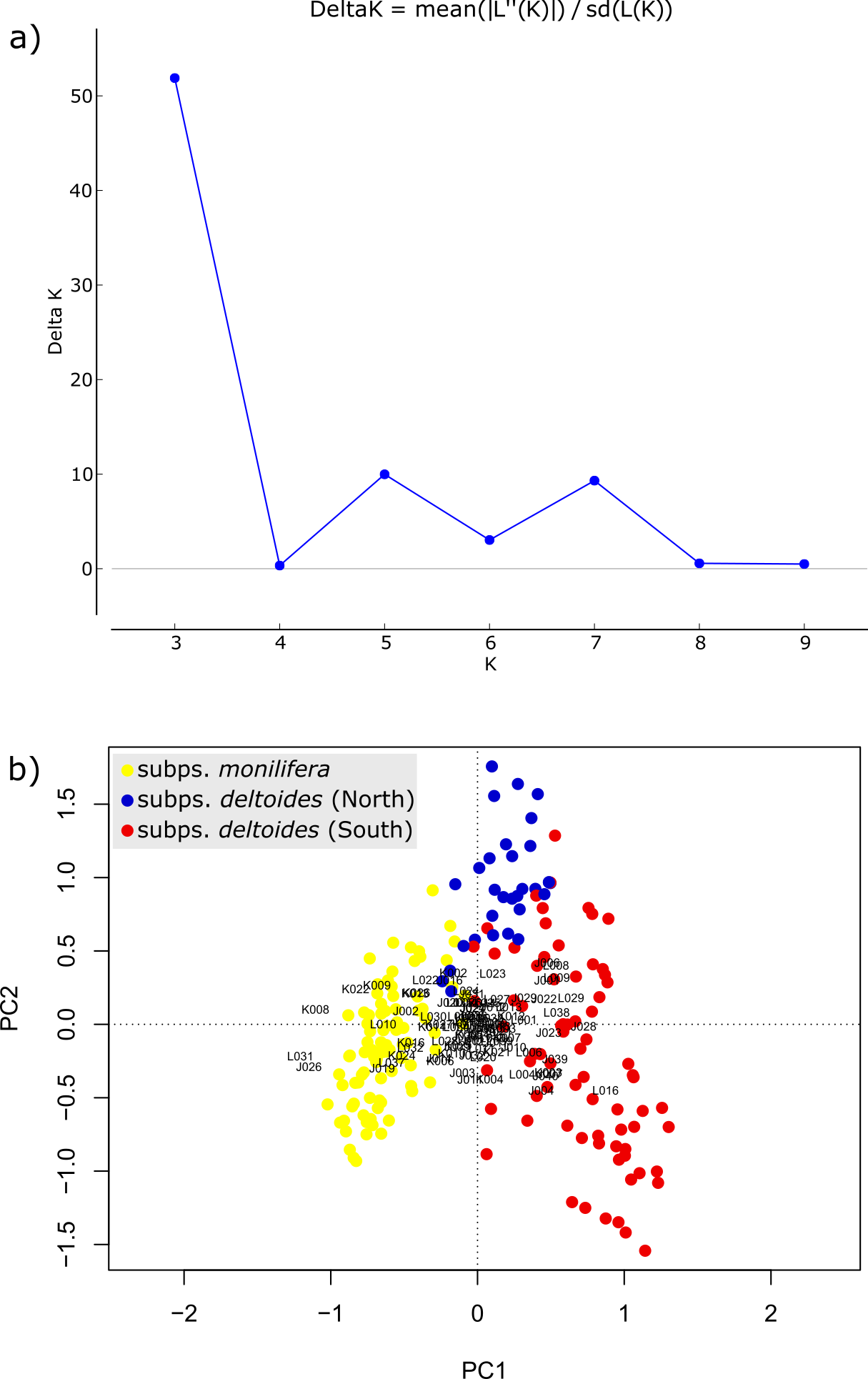
**

**Figure S4.** Credible intervals (0.9) obtained by Structure for the 946 *P. deltoides* individuals. Colors of the points and intervals in each graphic correspond to the three Q-values calculated for each group: yellow=subsp. *monilifera*, red=southern subsp. *deltoides*, blue= northeastern subsp. *deltoides*. Grey lines refer to the credible intervals from the two non-focal Structure groups in each graphic. The left part of the graphic shows the individuals that are considered as “pure” (i.e. with a Structure Q-value > 0.667) and for which there is low or no overlap between the three credible intervals calculated. a) Individuals are arranged in decreasing order for Q-values associated to the subsp. *monilifera* group. b) Individuals are arranged in decreasing order for Q-values associated to the southern subsp. *deltoides* group. c) Individuals are arranged in decreasing order for Q-values associated to the northeastern subsp. *deltoides* group.

**
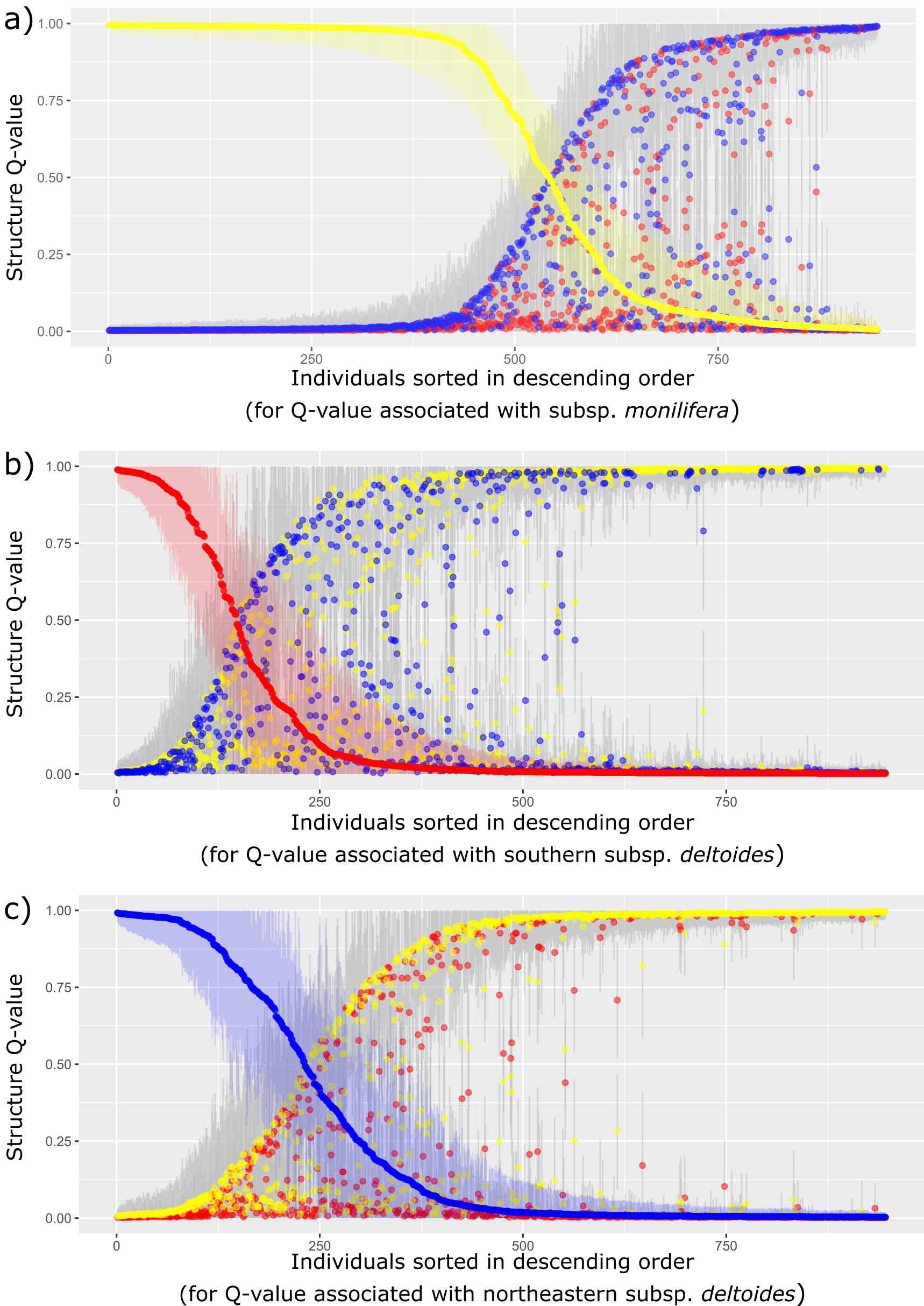
**

**Figure S5** Histograms presenting the partitioning variance of genomic variability over all environmental variables (separated in different categories) for the entire sample and the different groups delineated in this study. Only significant categories are presented (*p* < 0.05). The interaction category corresponds to the interaction between all variables.


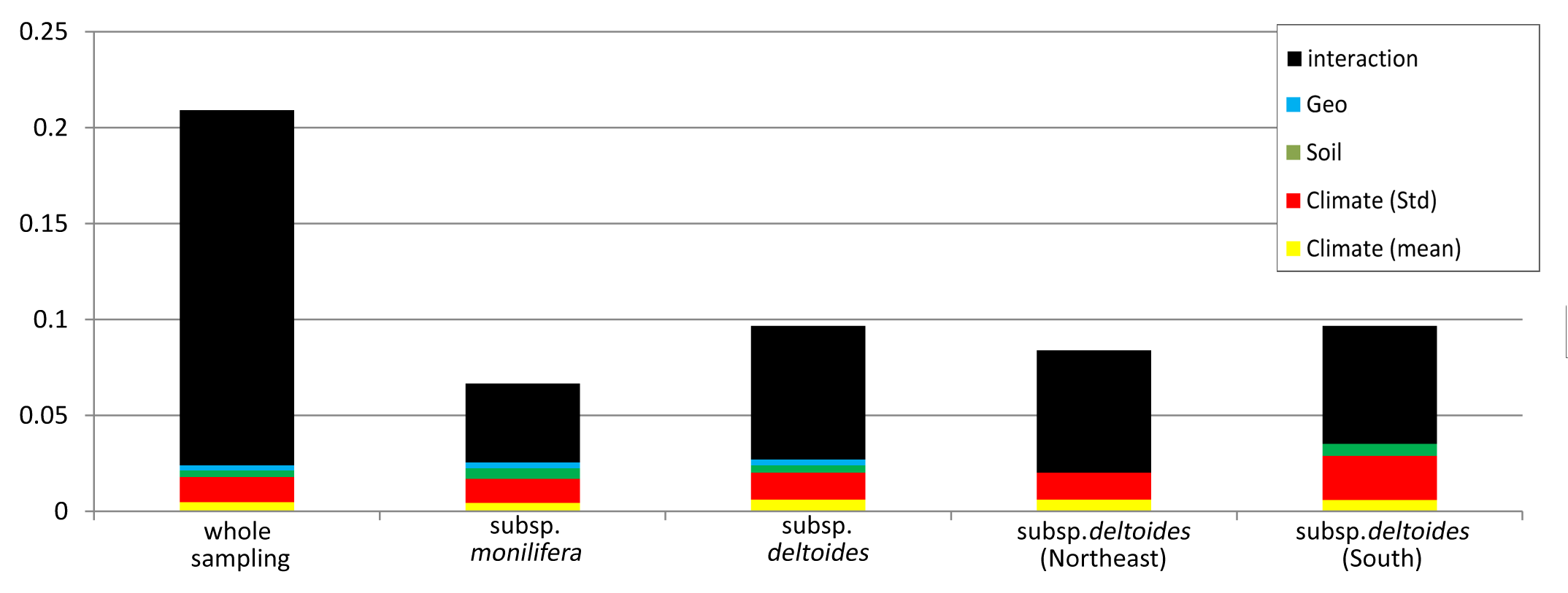


**Figure S6** Boxplots representing environmental variation associated with different genotypes (0=AA, 1=AB, 2=BB) for each marker identified by the LFMM analysis.


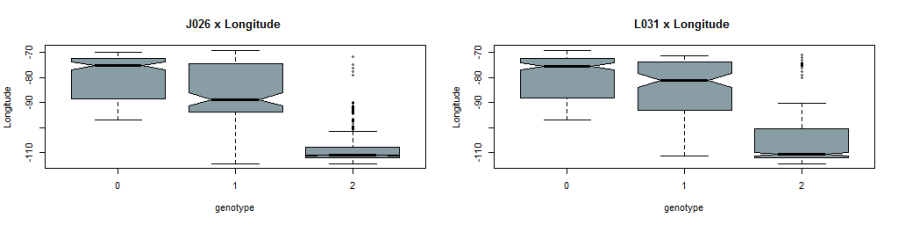


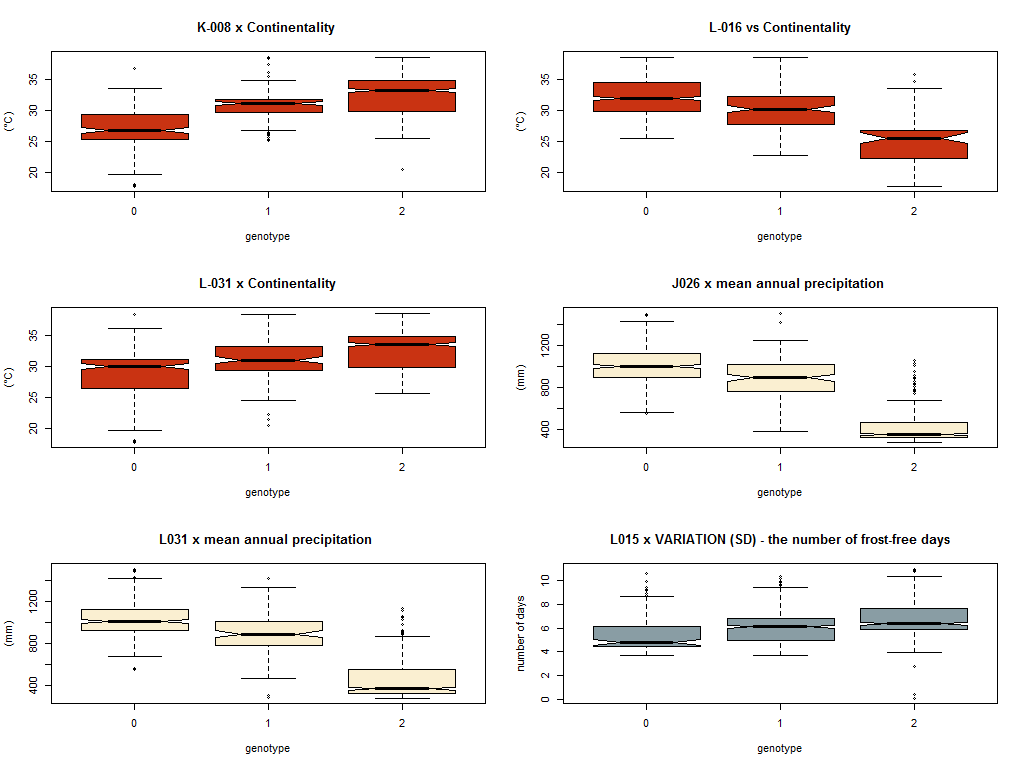


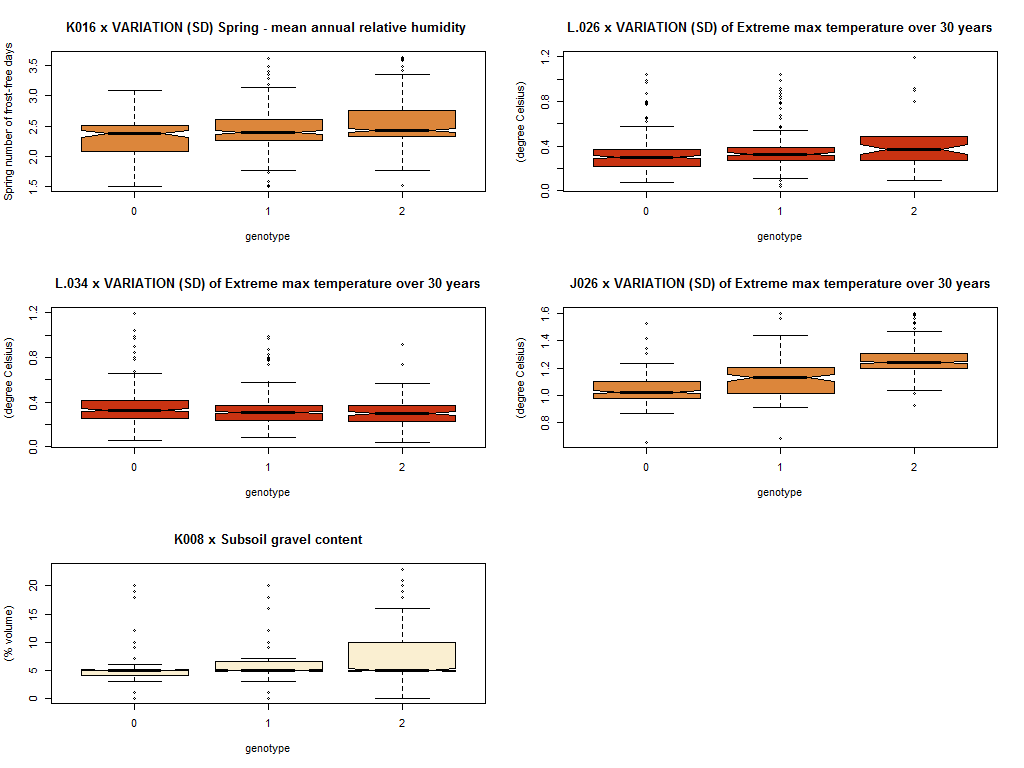


**Figure S7** Manhattan-like plots presenting the *F*_st_ calculated for each SNP between each pool vs the three others (b, c, d, e) and among the three “pure” pools (i.e. without the admixed pool) (a). The red line in each graph corresponds to the mean *F*_st_ calculated for each SNP. The blue line corresponds to the 99th percentile of SNP *F*_st_ calculated among the three most distinct groups (i.e. without the admixed pool), i.e. 0.466. Highlighted SNPs correspond to the SNPs identified
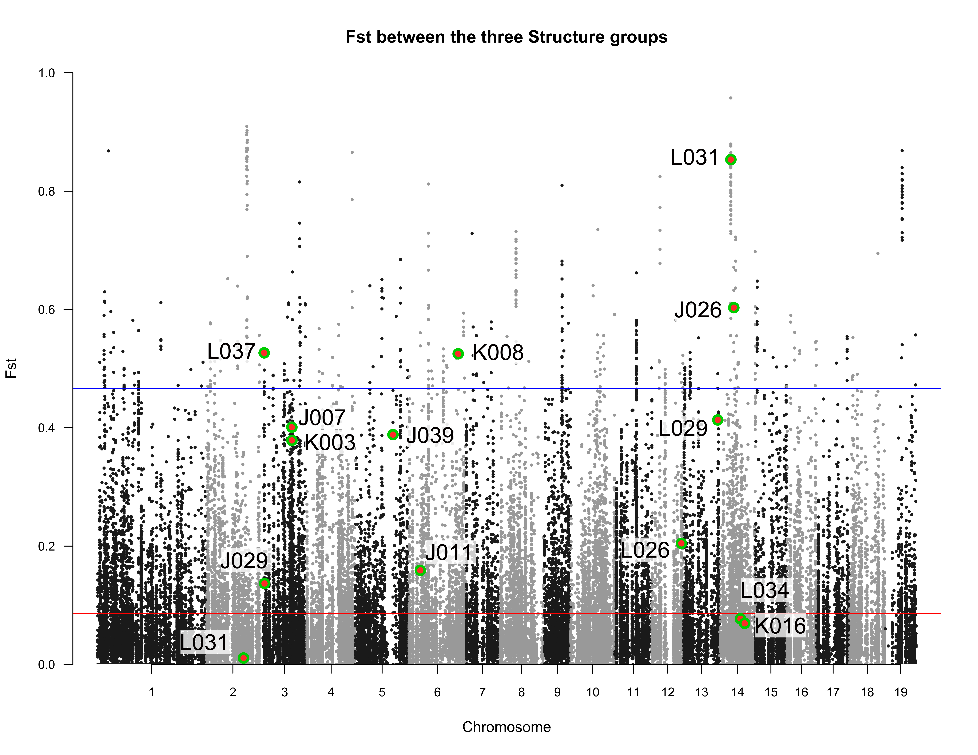
by one of the two approaches used in the present study (FDist or LFMM analyses).

a)


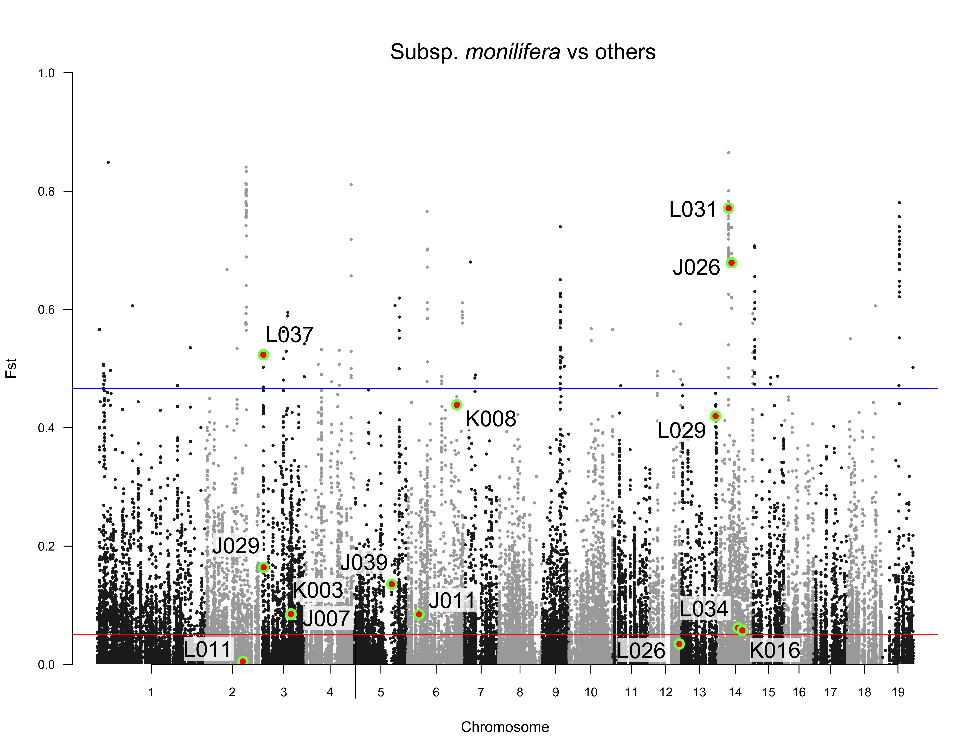


b)


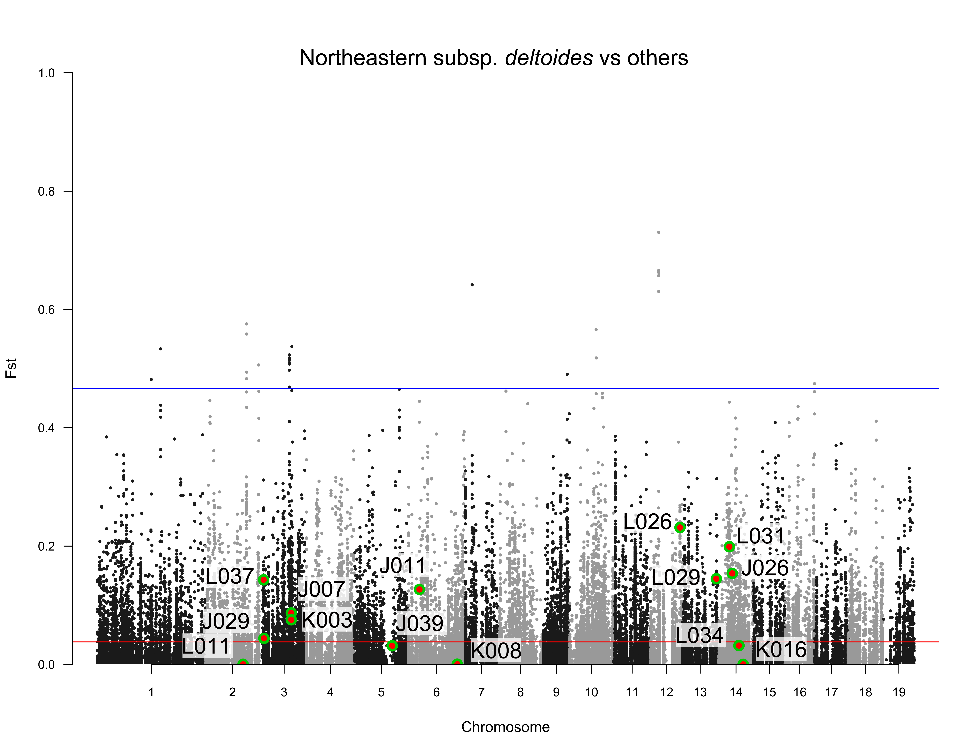


c)


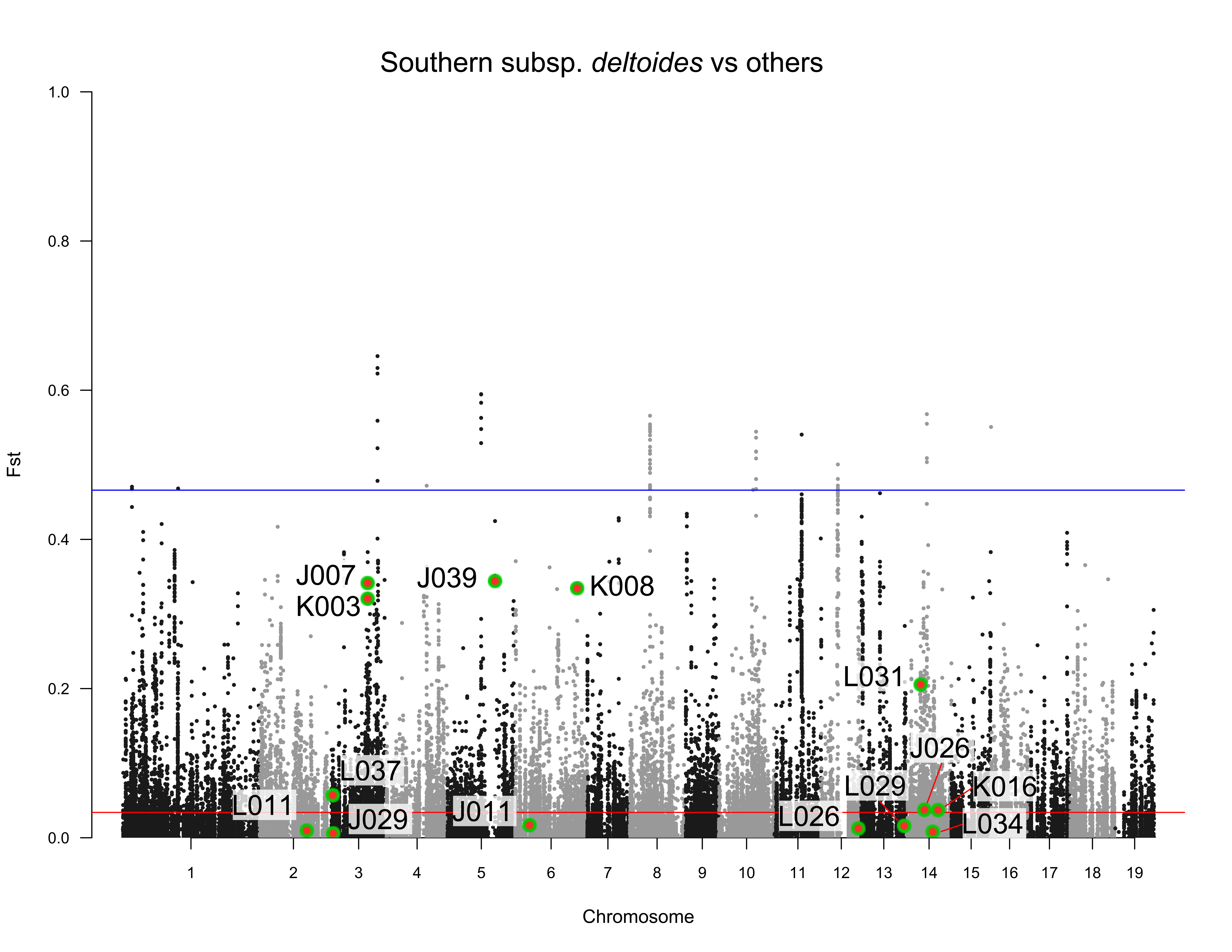


d)


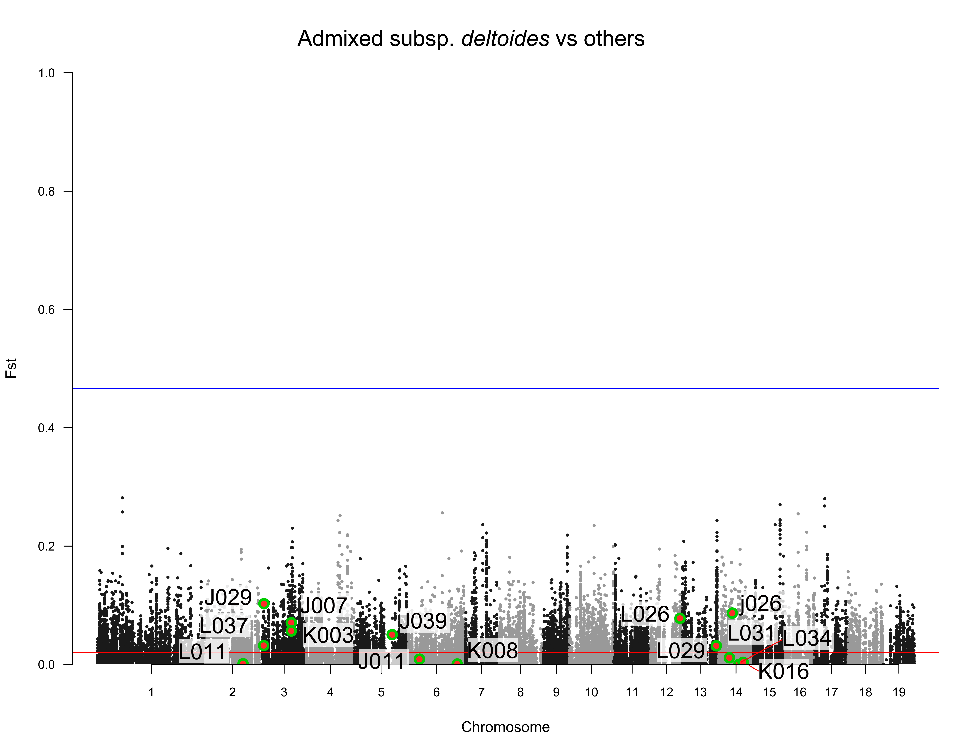
e)
