# Supplementary material for: Going with the flow: Intraspecific variation may act as a natural ally to counterbalance the impacts of global change for the riparian species Populus deltoides
Source: Evol Appl. 2019 Sep 20;13(1):176–94. doi: 10.1111/eva.12854 (PMC6935597; doi:10.1111/eva.12854)
Supplement: Supplementary file 3 [file EVA-13-176-s003.pdf]

Appendix 1.

Elevation, climate (mean and standard deviation) and soil variables at the scale of the *P. deltoides* natural distribution. Color dots represents the membership of individuals to each of the genetic groups identified in the study (yellow= ssp. *monilifera*, red=southern ssp. *deltoides*, blue= northern ssp. *deltoides*, stars= admixed membership, i.e. Structure Q-values < 0.9 for each of the three groups)

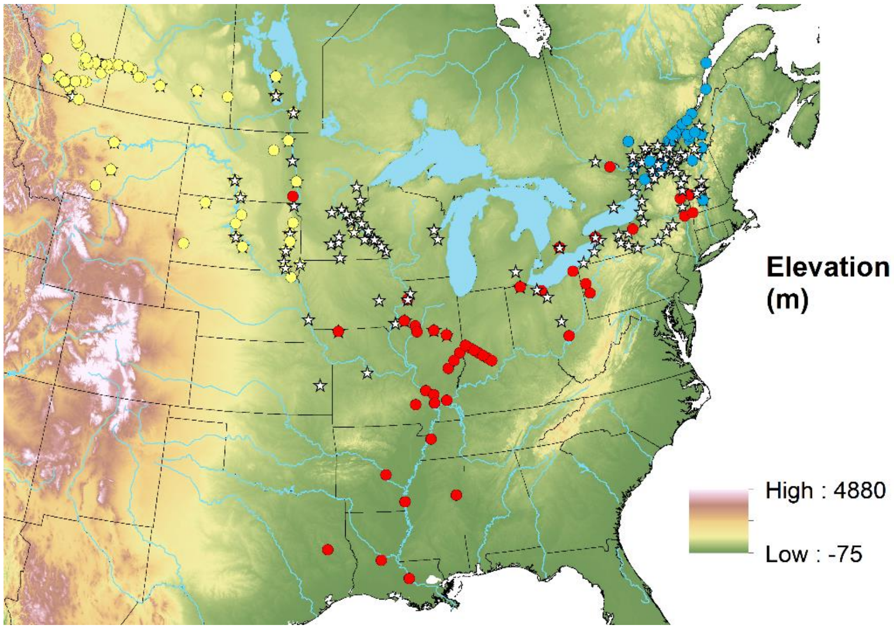

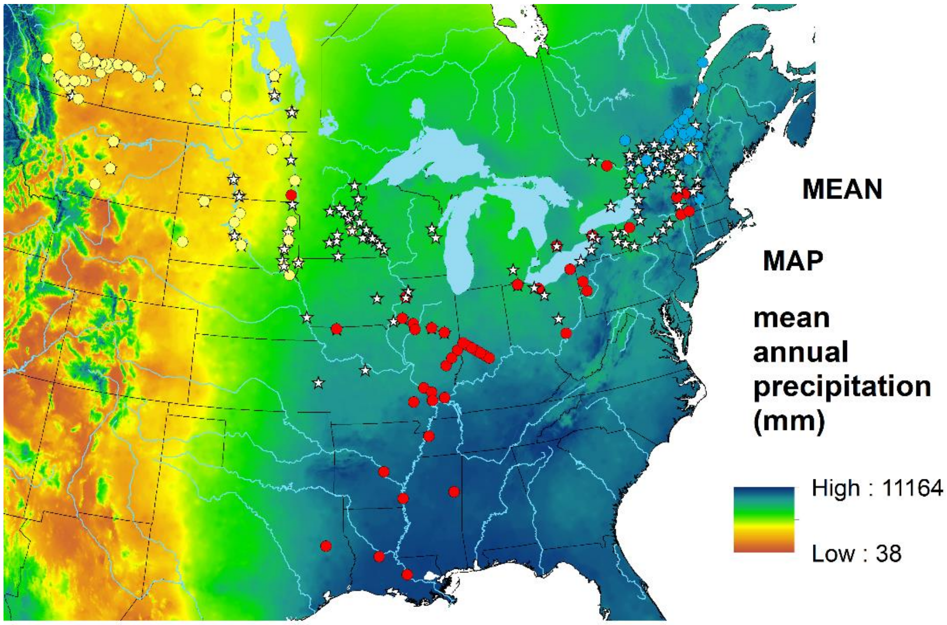

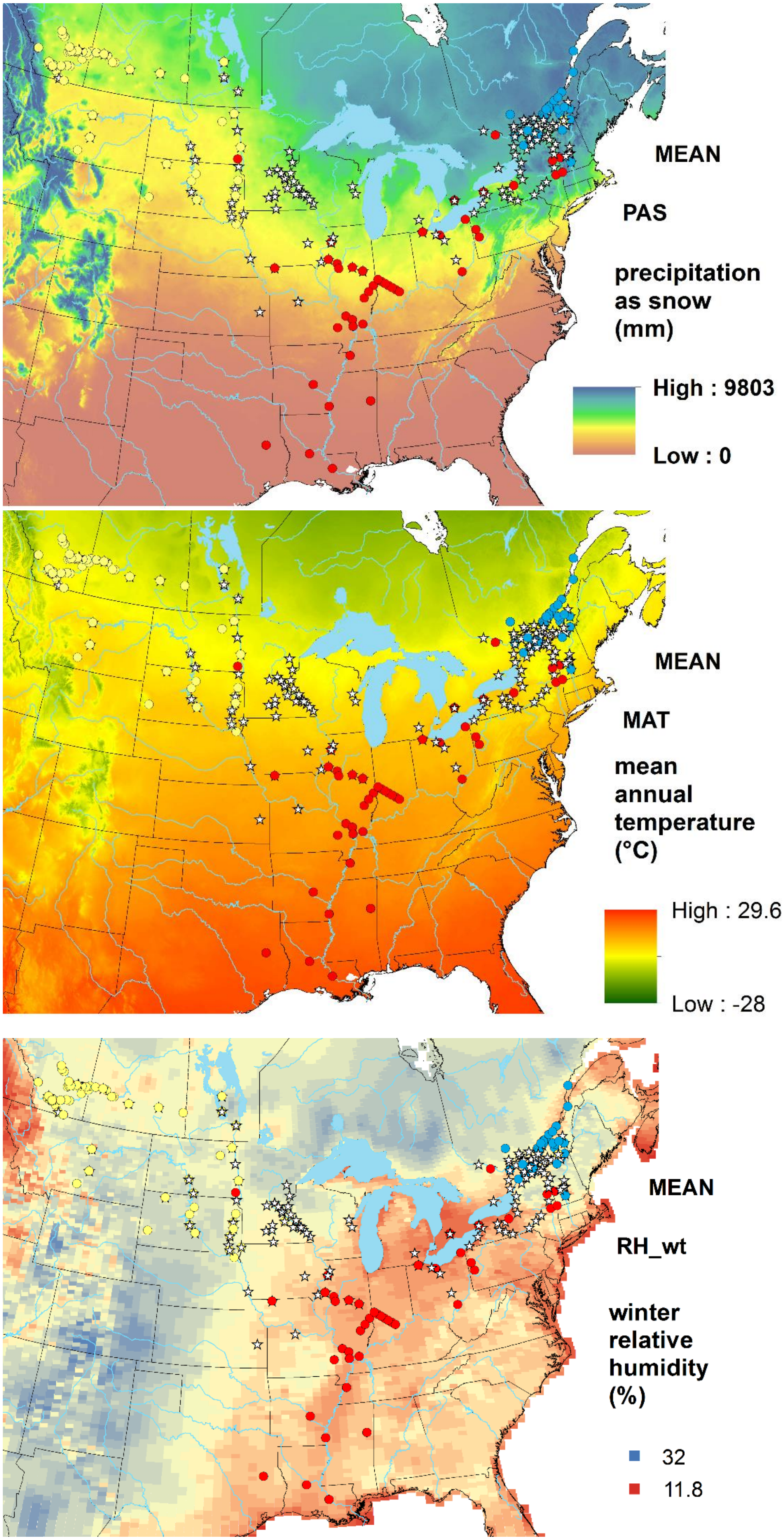

MEAN
PAS
precipitation as snow (mm)
High : 9803
Low : 0
MEAN
MAT
mean annual temperature (°C)
High : 29.6
Low : -28
MEAN
RH_wt
winter relative humidity (%)
32
11.8

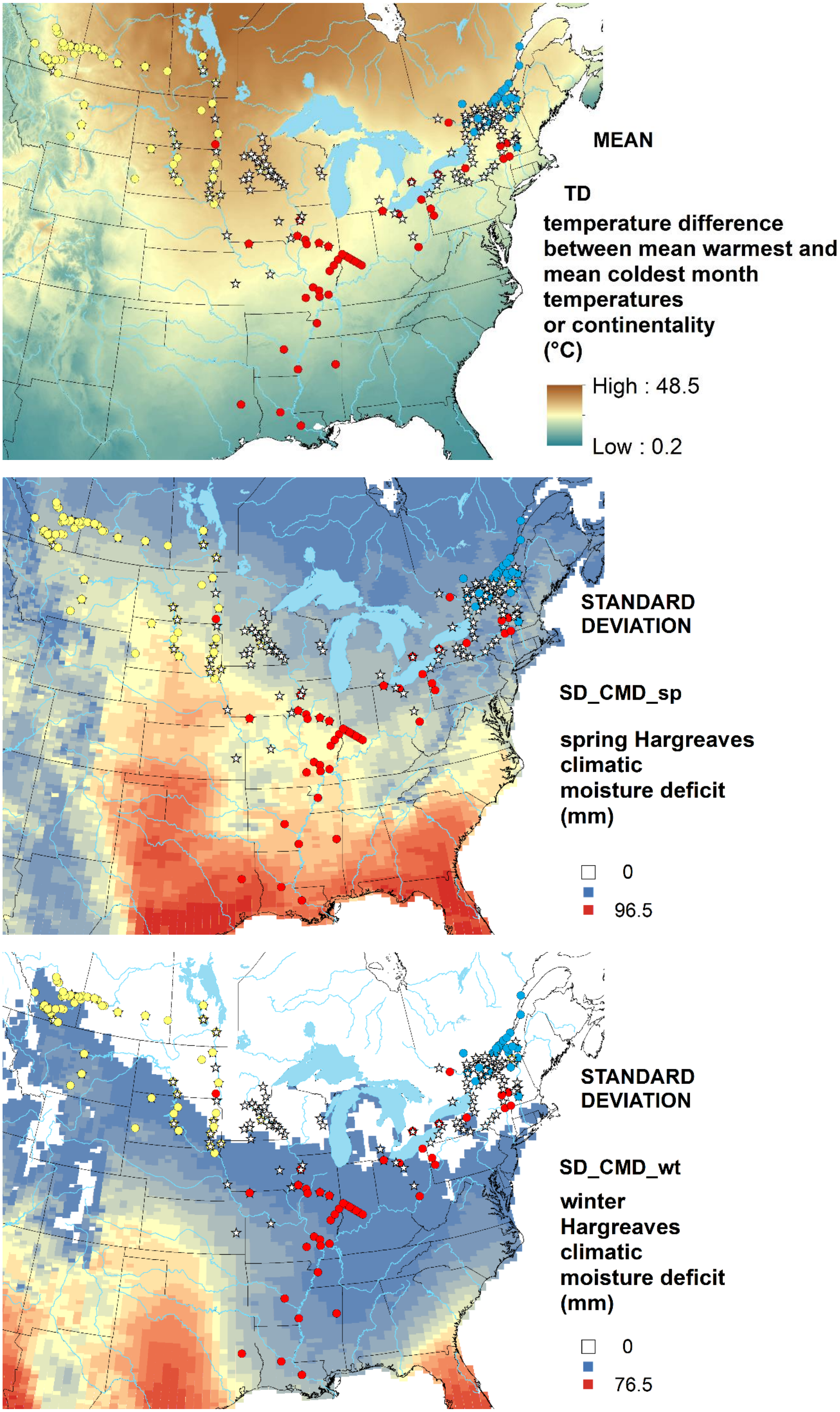
MEAN
TD
temperature difference
between mean warmest and
mean coldest month
temperatures
or continentality
(°C)
High : 48.5
Low : 0.2
STANDARD
DEVIATION
SD_CMD_sp
spring Hargreaves
climatic
moisture deficit
(mm)
0
96.5
STANDARD
DEVIATION
SD_CMD_wt
winter
Hargreaves
climatic
moisture deficit
(mm)
0
76.5

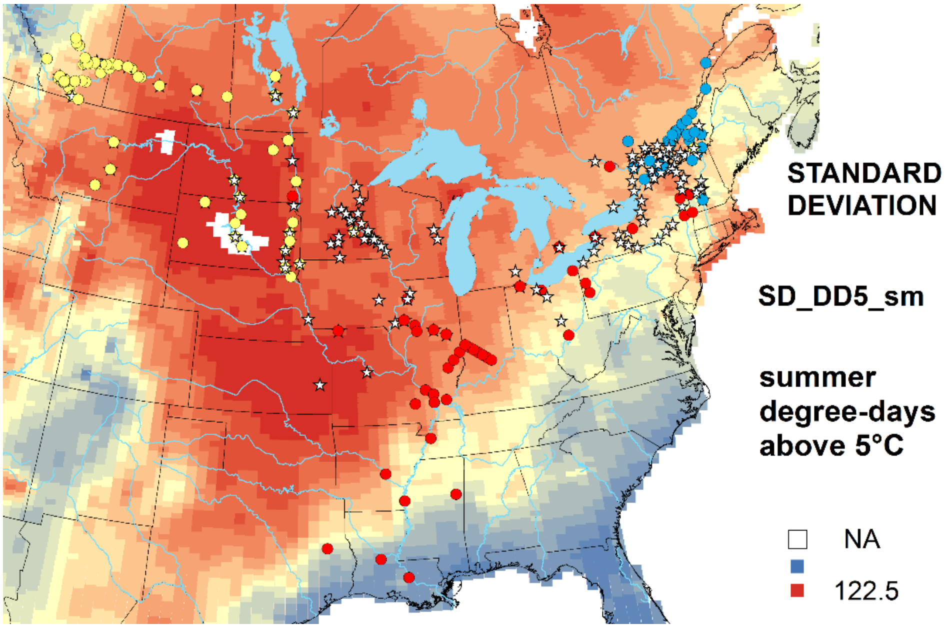

STANDARD
DEVIATION

SD_DD5_sm

summer
degree-days
above 5°C

☐ NA
■
■ 122.5

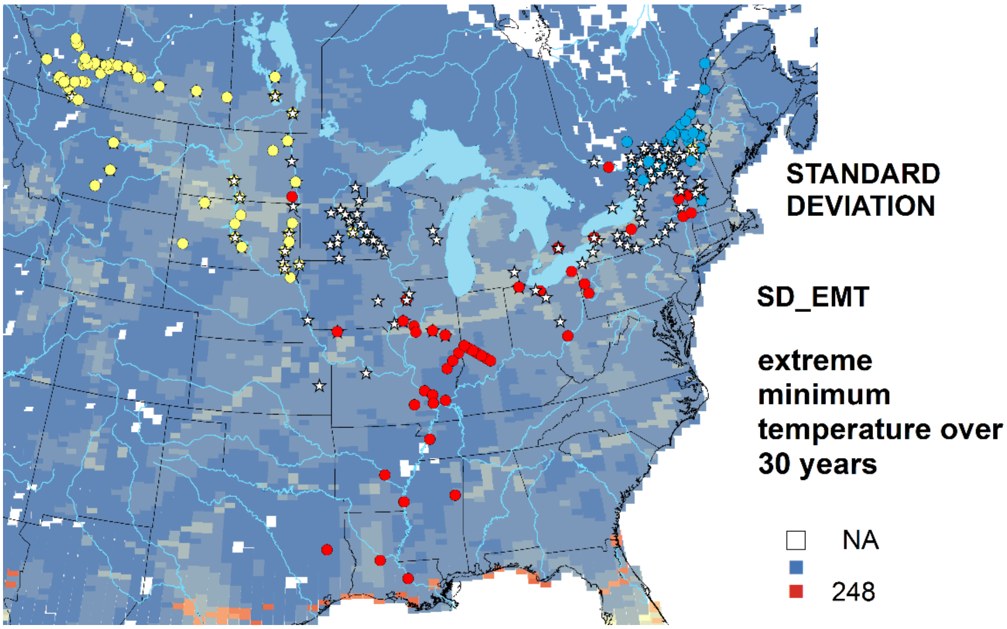

STANDARD
DEVIATION

SD_EMT

extreme
minimum
temperature over
30 years

☐ NA
■
■ 248

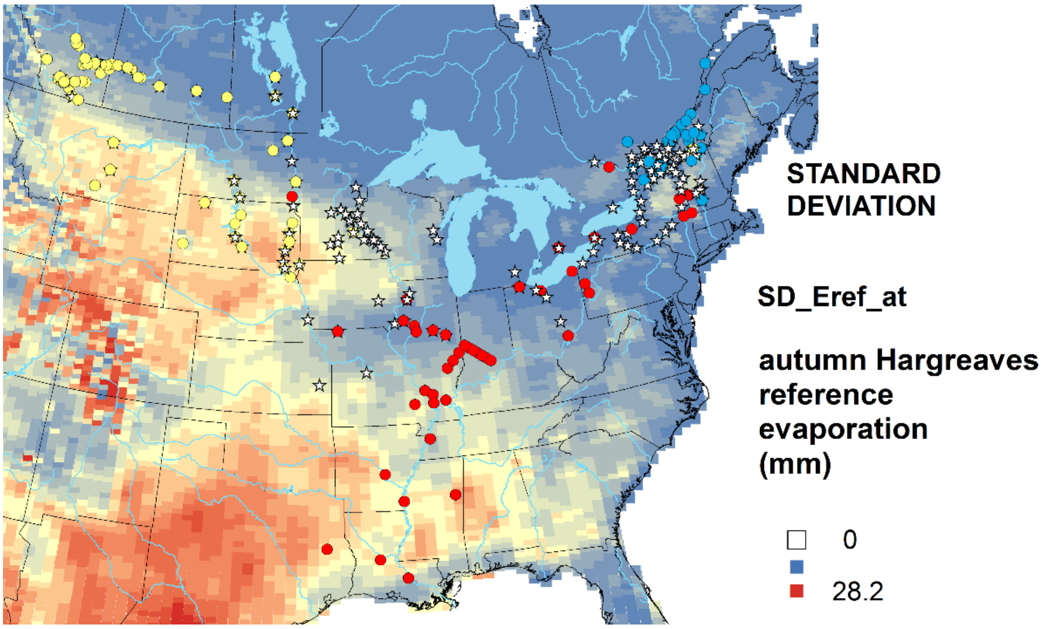

STANDARD
DEVIATION

SD_Eref_at

autumn Hargreaves
reference
evaporation
(mm)

☐ 0
■
■ 28.2

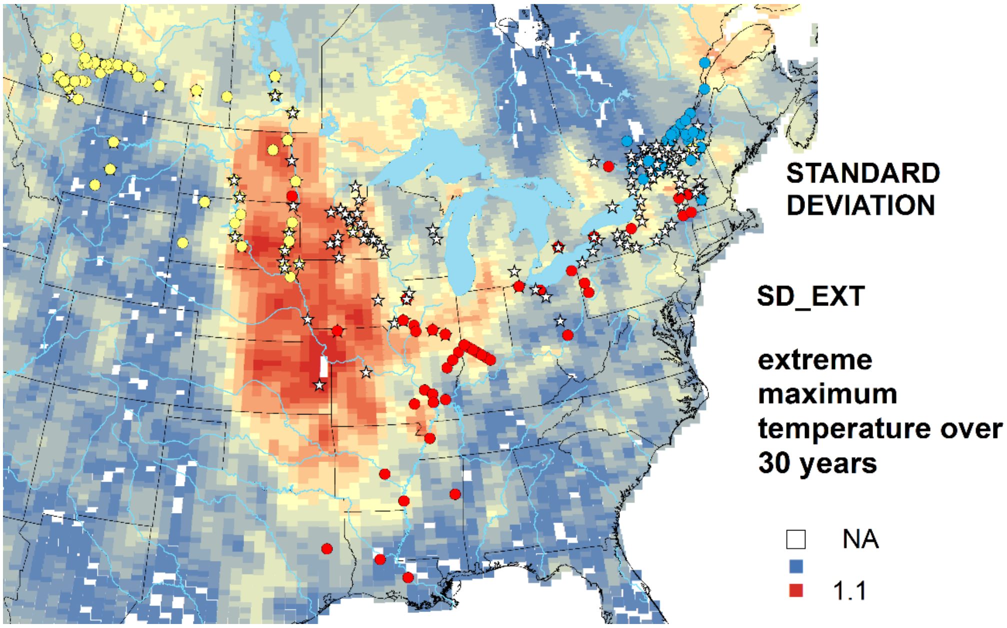

STANDARD DEVIATION

SD_EXT

extreme maximum temperature over 30 years

NA

1.1

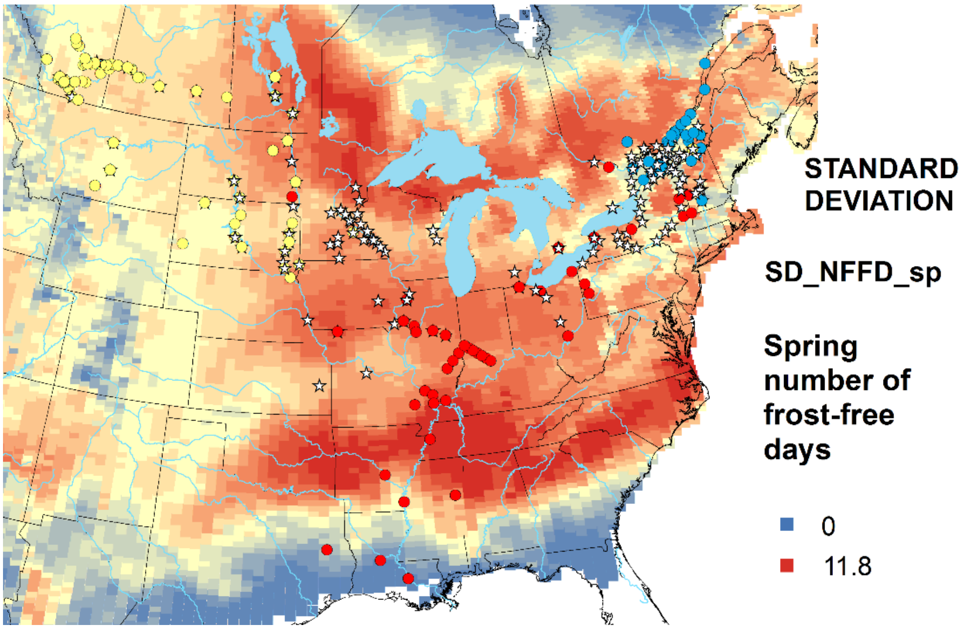

STANDARD DEVIATION

SD_NFFD_sp

Spring number of frost-free days

0

11.8

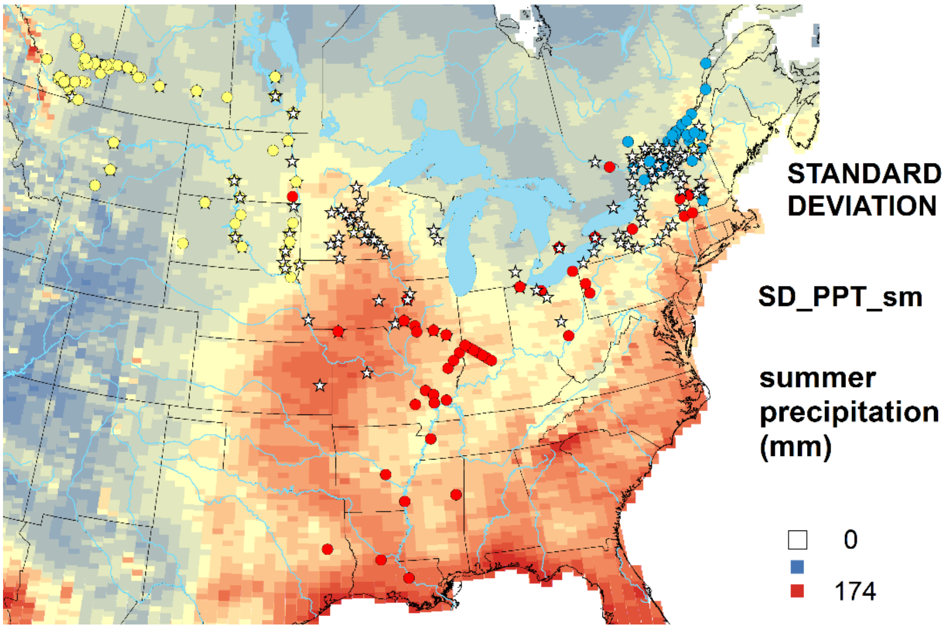

STANDARD DEVIATION

SD_PPT_sm

summer precipitation (mm)

0

174

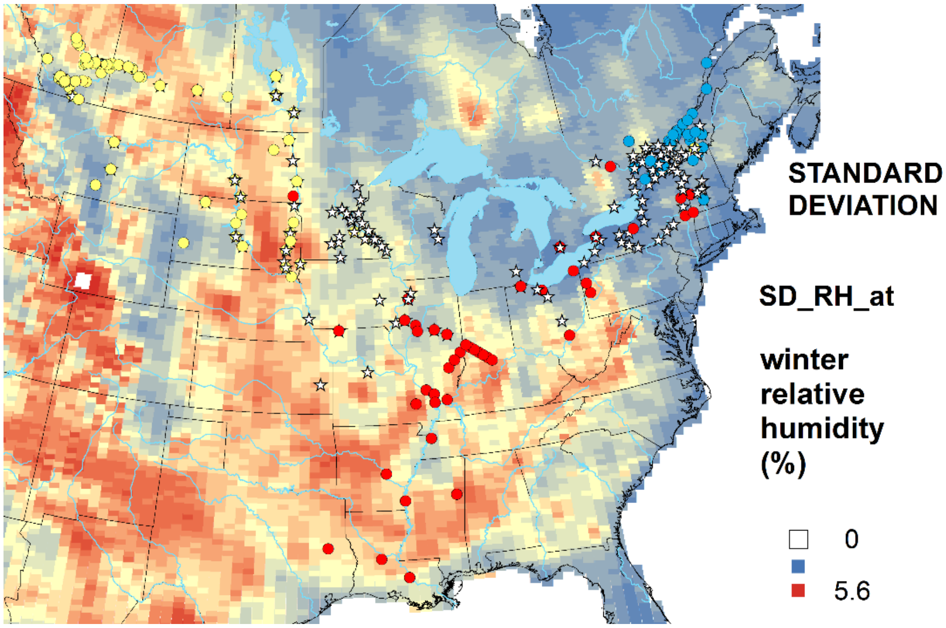
STANDARD
DEVIATION
SD_RH_at
winter
relative
humidity
(%)
0
5.6

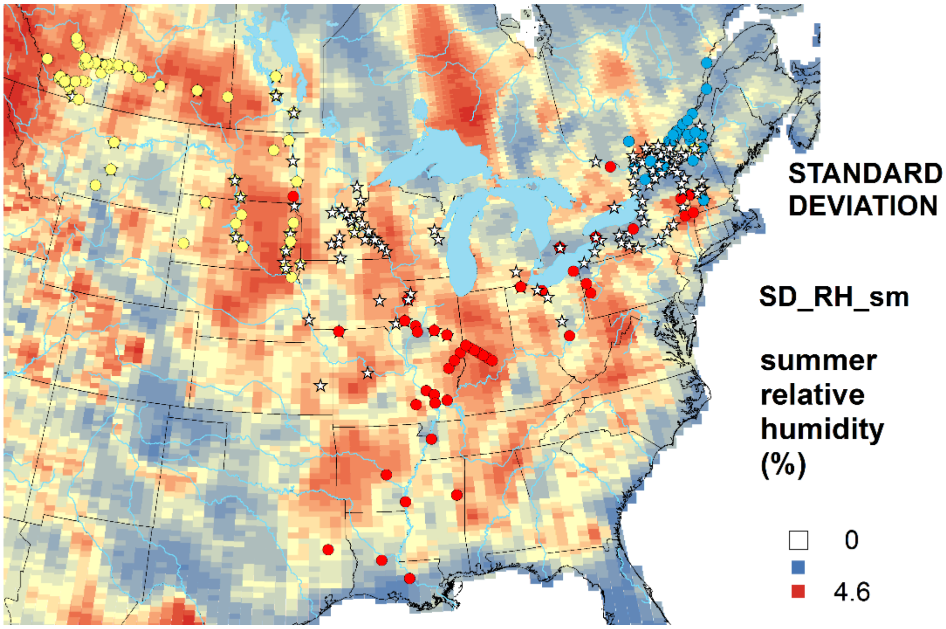
STANDARD
DEVIATION
SD_RH_sm
summer
relative
humidity
(%)
0
4.6

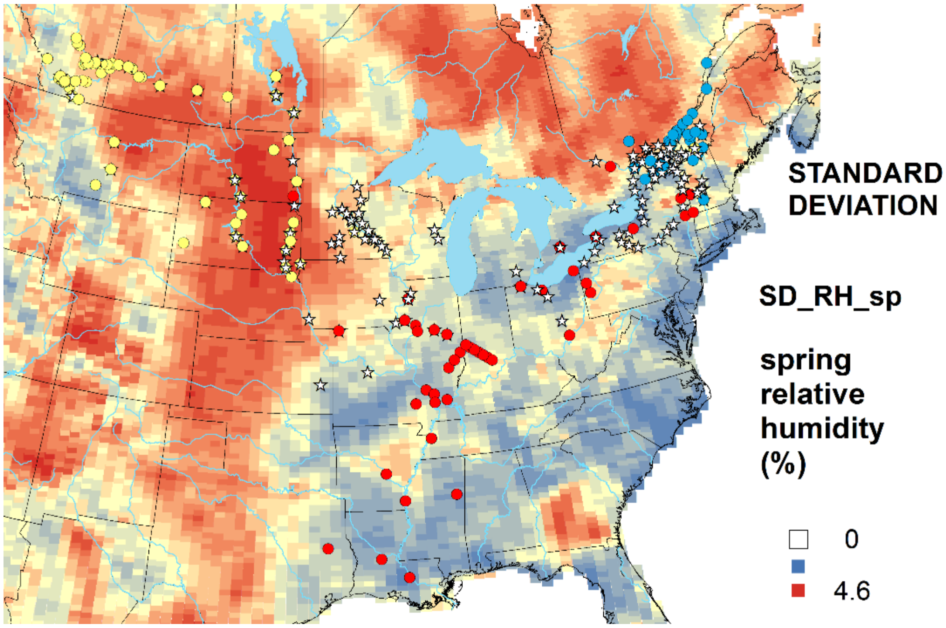
STANDARD
DEVIATION
SD_RH_sp
spring
relative
humidity
(%)
0
4.6

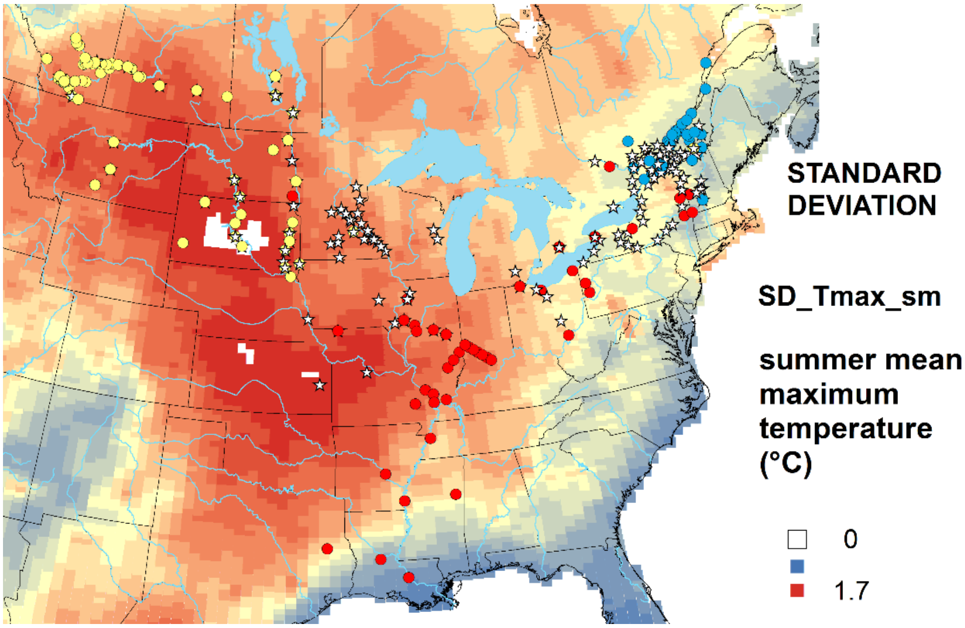

STANDARD DEVIATION

SD_Tmax_sm

summer mean maximum temperature (°C)

0

1.7

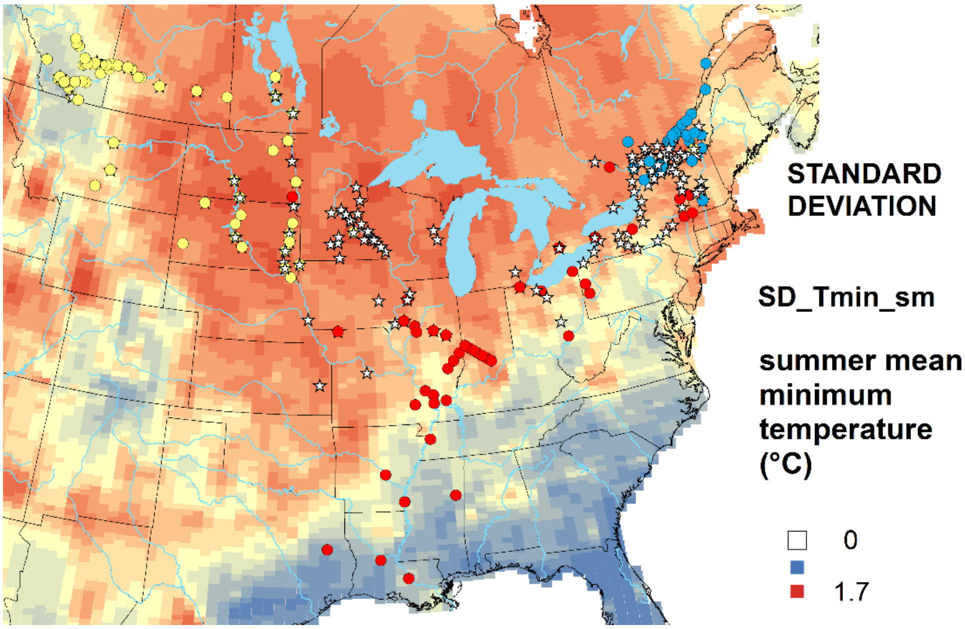

STANDARD DEVIATION

SD_Tmin_sm

summer mean minimum temperature (°C)

0

1.7

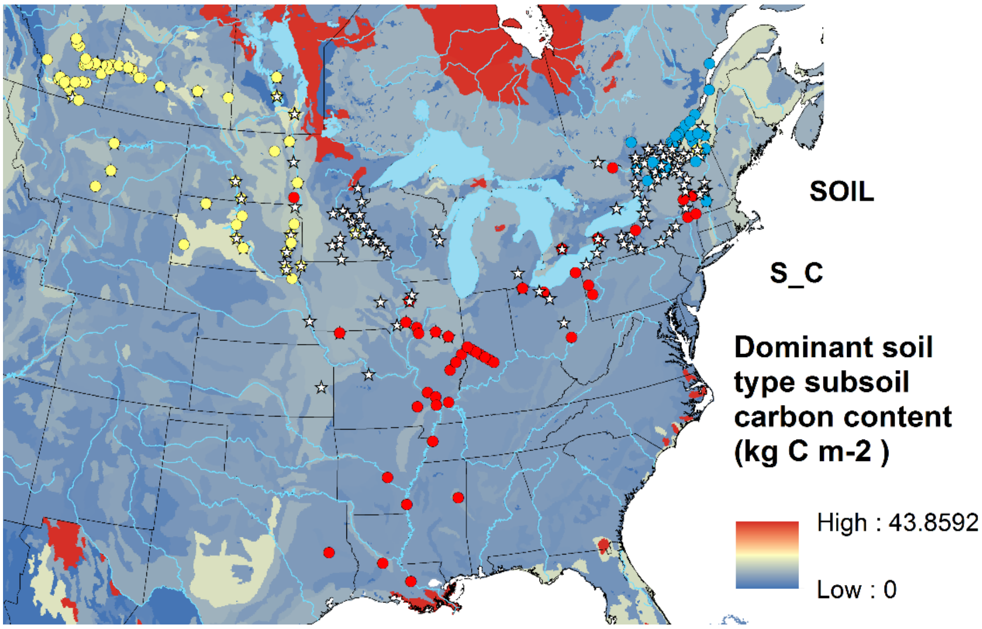

SOIL

S_C

Dominant soil type subsoil carbon content (kg C m-2)

High : 43.8592

Low : 0

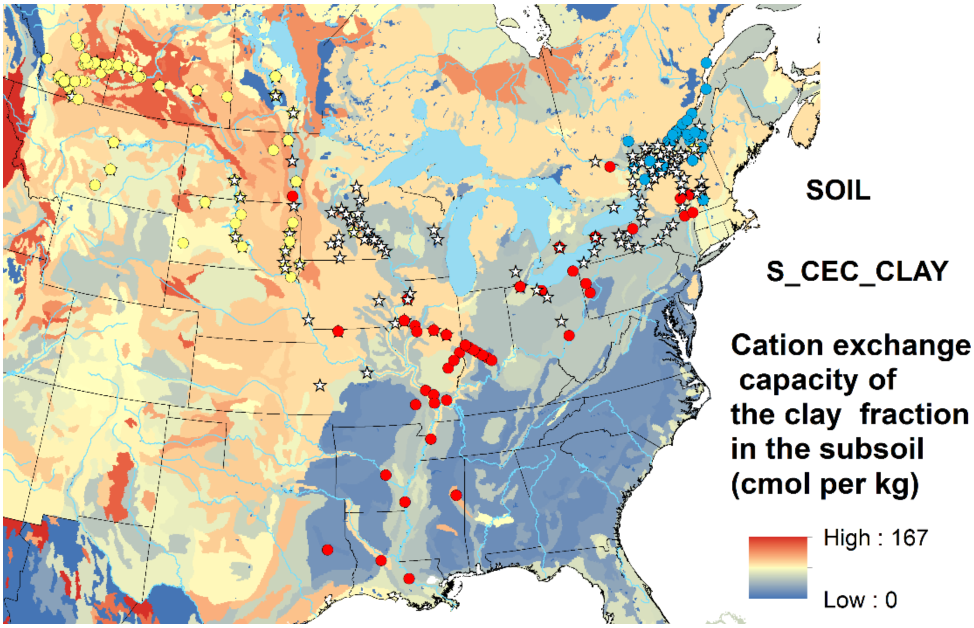

SOIL

S_CEC_CLAY

Cation exchange capacity of the clay fraction in the subsoil (cmol per kg)

High : 167

Low : 0

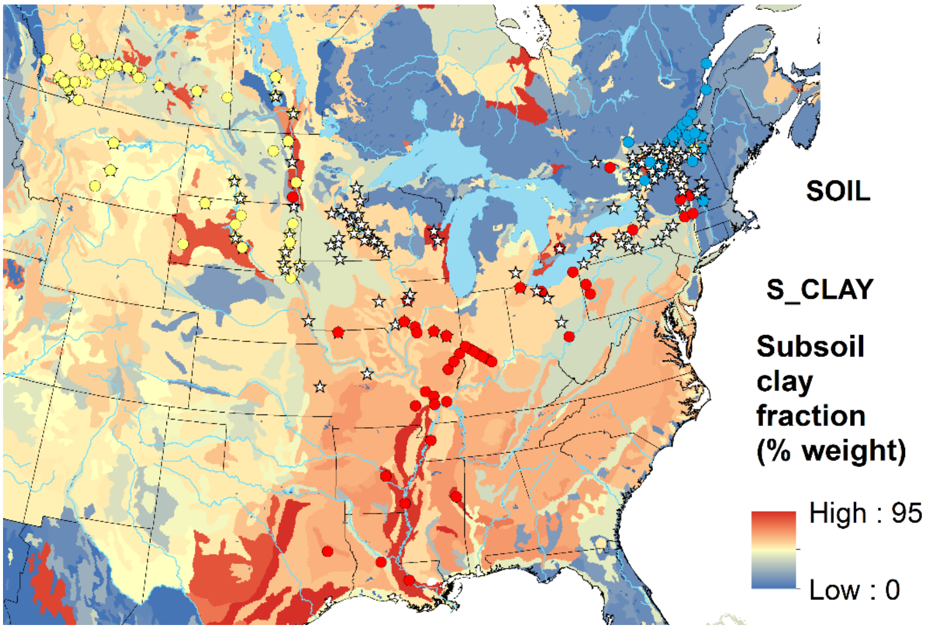

SOIL

S_CLAY

Subsoil clay fraction (% weight)

High : 95

Low : 0

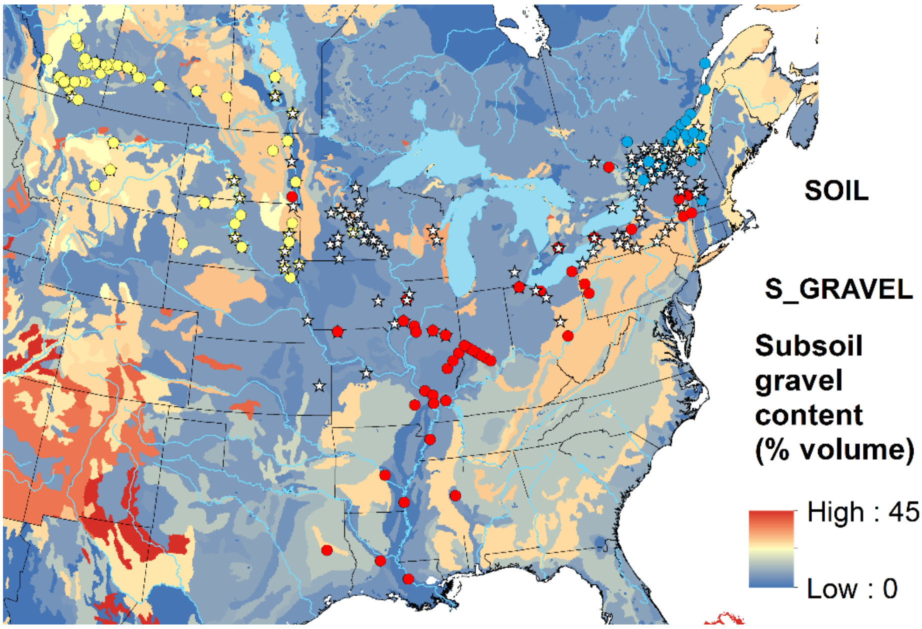

SOIL

S_GRAVEL

Subsoil gravel content (% volume)

High : 45

Low : 0

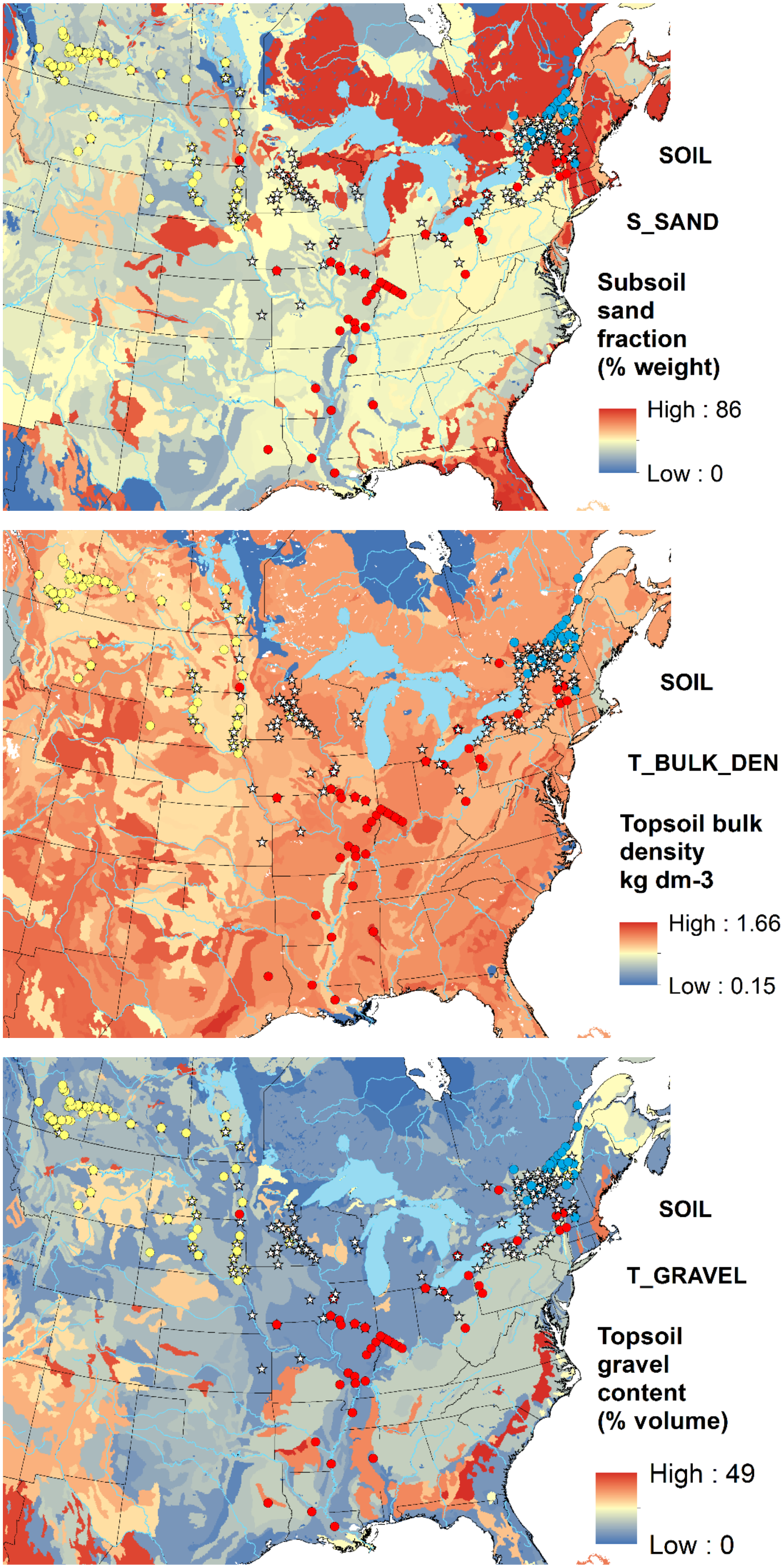
SOIL
S_SAND
Subsoil sand fraction (% weight)
High : 86
Low : 0
SOIL
T_BULK_DEN
Topsoil bulk density kg dm-3
High : 1.66
Low : 0.15
SOIL
T_GRAVEL
Topsoil gravel content (% volume)
High : 49
Low : 0

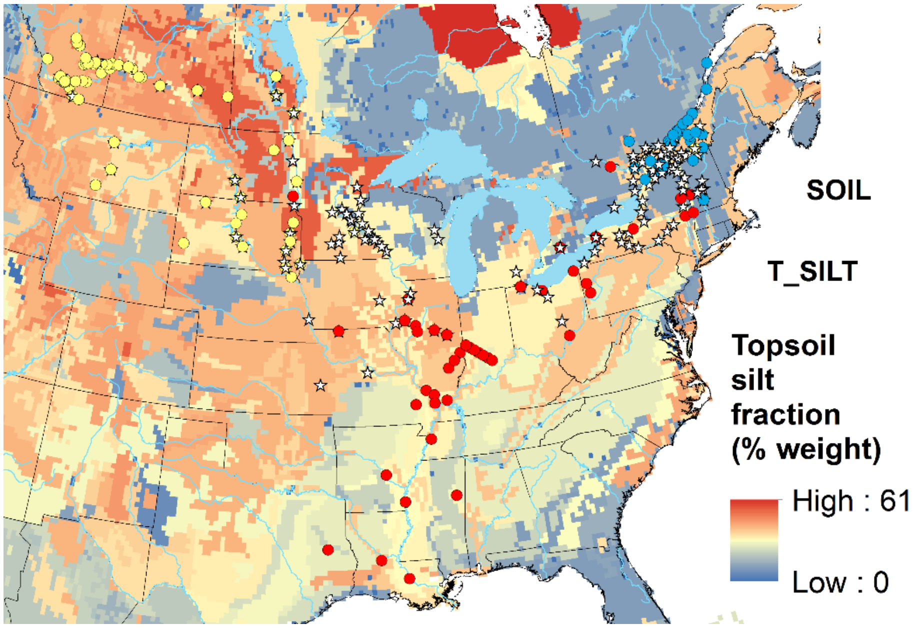
SOIL
T_SILT
Topsoil
silt
fraction
(% weight)
High : 61
Low : 0
